# Bioinspired phosphate homeostasis for atomically precise regulation of silver nanoclusters

**DOI:** 10.1093/nsr/nwaf183

**Published:** 2025-05-10

**Authors:** Wei-Dan Si, Lu-Yang Xing, Álvaro Muñoz-Castro, Chengkai Zhang, Bao-Liang Han, Jian-Long Zhou, Zhi Wang, Chen-Ho Tung, Di Sun

**Affiliations:** School of Chemistry and Chemical Engineering, State Key Laboratory of Crystal Materials, Shandong University, Jinan 250100, China; School of Chemistry and Chemical Engineering, State Key Laboratory of Crystal Materials, Shandong University, Jinan 250100, China; Facultad de Ingeniería, Arquitectura y Diseño, Universidad San Sebastián, Santiago 8420524, Chile; School of Chemistry and Chemical Engineering, State Key Laboratory of Crystal Materials, Shandong University, Jinan 250100, China; School of Chemistry and Chemical Engineering, State Key Laboratory of Crystal Materials, Shandong University, Jinan 250100, China; School of Chemistry and Chemical Engineering, State Key Laboratory of Crystal Materials, Shandong University, Jinan 250100, China; School of Chemistry and Chemical Engineering, State Key Laboratory of Crystal Materials, Shandong University, Jinan 250100, China; School of Chemistry and Chemical Engineering, State Key Laboratory of Crystal Materials, Shandong University, Jinan 250100, China; School of Chemistry and Chemical Engineering, State Key Laboratory of Crystal Materials, Shandong University, Jinan 250100, China

**Keywords:** phosphate balance, dynamic assembly, high-nuclearity silver clusters, anion template

## Abstract

The dynamic dissociation equilibrium of phosphate in living organisms plays a crucial role in maintaining the balance necessary for sustaining life. In the field of metal clusters, [H_3−_*_x_*PO_4_]*^x^*^−^ (*x* = 1–3) anions also serve as effective templates for constructing silver clusters, with their innate structural flexibility bringing tremendous promise for structural regulation. However, current understanding of the effects of phosphate balance on the dynamic assembly of high-nuclearity silver clusters (metal atom number > 100) remains limited. In this study, we first demonstrate that different forms of phosphates (orthophosphate, hydrogen phosphate and dihydrogen phosphate) can controllably provide tetrahedral PO_4_^3−^ oxyanions in the basic environment, thereby directing the structural evolution of silver clusters. A multilayered, rosette-shaped 104-nuclei silver nanocluster (**Ag104a**) is successfully isolated by utilizing Na_3_PO_4_/Na_2_HPO_4_ as the PO_4_^3−^ source. This unique structure features a silver-containing (PO_4_)@Ag_4_@(PO_4_)_12_ template layer, enveloped by an outer Ag_100_ shell composed of an Ag_72_ garland and two Ag_14_ units. Notably, **Ag104a** represents the silver alkynyl cluster with the highest number of encapsulated tetrahedral anions to date. In contrast, using NaH_2_PO_4_ results in the formation of a different co-crystallized silver cluster: **Ag104b·Ag108a**. Time-dependent ^31^P nuclear magnetic resonance analysis on the reaction solution reflected the different release rates of PO_4_^3−^ anions, which can affect the assembly of silver clusters. This work not only makes a significant advancement in the structural regulation of high-nuclearity silver clusters by phosphates, but also offers valuable insights into the intricate interplay between phosphate balance and the dynamic assembly of silver clusters.

## Teaser

This study demonstrates how different forms of phosphates regulate the assembly processes of high-nuclearity (metal atom number >100) silver clusters.

## INTRODUCTION

The burgeoning trajectory of interest in the field of cluster chemistry is currently captivating the scientific community, expanding the structural diversity of metal nanoclusters for next-generation functional materials [1–10]. Over the past two decades, one of the most important members in the cluster family—silver cluster—has emerged as a focal point of significant interest, garnering attention owing to its diverse structure, properties and promising applications [11–18]. In particular, high-nuclearity silver clusters (metal atom number > 100) boast charming aesthetic structures, rendering them one of the pursuits of synthetic chemists in a challenging field [19–33]. Various ligands have been utilized for stabilizing large metallic skeletons, including phosphine [19,20], thiolate [21–26], alkynyl [27–29], halides [30–32], *p*-tert-butylthiacalix[4]arene [33] and so on. Anion templates, distinguished by their multiple negative charges and diverse geometries, play a vital role in mediating the size, structure and property of silver clusters from within [34]. The anion-template synthetic strategy is recognized as a popular route for the controlled assembly of these clusters [35]. Compared with the simple ions (Cl^−^, S^2−^) [36–38], oxyanion templates with rich geometric shapes (such as triangular NO_3_^−^, CO_3_^2−^; tetrahedral ^99^TcO_4_^−^, SO_4_^2−^, CrO_4_^2−^, MoO_4_^2−^, PO_4_^3−^, AsO_4_^3−^, VO_4_^3−^; octahedral TeO_6_^6−^ and higher bulk polyoxometalates) have larger size and higher negative charge, providing more coordination sites for the silver atoms and thus beneficial for constructing high-nuclearity silver clusters [39–46]. Although diverse anion-templated silver clusters have been successfully isolated, the synthesis of silver clusters with a total metal count of >100 remains a rare achievement. Notable examples include: [Ag_102_S_6_(PO_4_)_8_(CyS)_30_(H_2_PO_4_)_6_(HPO_4_)_6_(CF_3_COO)_18_]·6MeOH·6H_2_O [47], [Ag_12_@Ag_20_@(KPO_4_)_10_@Ag_70_(^*t*^BuPhS)_60_(CF_3_COO)_10_(DMF)_2_] [48] and (H_3_O)_2_[(SO_4_)_36_S_22_@Ag_192_(CyS)_66_(NO_3_)_12_]·4CH_3_OH [26], all of which contain tetrahedral anion templates by using NaH_2_PO_4_, KH_2_PO_4_ and VOSO_4_·*x*H_2_O, respectively.

**Scheme 1.**
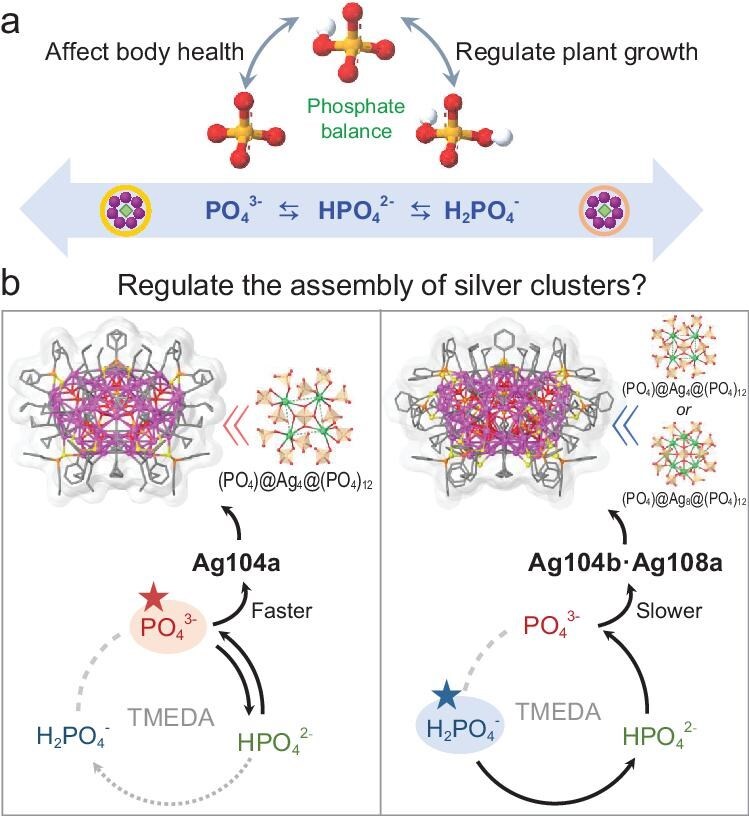
Synthetic conception. (a) Phosphate balance in nature. (b) Synthetic routes for **Ag104a** and **Ag104b·Ag108a**. Color legend: Ag, purple and green; P, orange; S, yellow; O, red; C, gray.

Phosphate salts, including Na_3_PO_4_, Na_2_HPO_4_ and NaH_2_PO_4_, play crucial roles in both health and the environment due to their active involvement in numerous industrial and biological processes (Scheme 1a). Corresponding phosphates, [H_3−_*_x_*PO_4_]*^x^*^−^ (*x* = 1−3), belong to the tetrahedral oxyanion family and are effective templates to construct high-nuclearity silver clusters [47–50]. Significant progress has been achieved in thiolate-protected silver clusters by using [H_3−_*_x_*PO_4_]*^x^*^−^ as templates, yet it remains unclear whether different protonated phosphate salts can markedly impact the structure, size and properties of high-nuclearity silver clusters. An essential consideration is that a complex acid–base dissociation balance can exist, such as PO_4_^3−^ ⇆ HPO_4_^2−^ ⇆ H_2_PO_4_^−^, resulting in multiple forms of phosphates with different charged states in solutions depending on the pH [51,52]. In our previous work, without the involvement of an acid or base, we isolated an Ag_102_ cluster containing three forms of phosphates when using NaH_2_PO_4_ as the incipient template agent, while the utilization of Na_3_PO_4_, a solely PO_4_^3−^ templated Ag_42_ cluster, was obtained in a similar reaction system [50]. However, in the presence of a base, what direction does the dynamic dissociation balance of [H_3−_*_x_*PO_4_]*^x^*^−^ proceed toward during the synthesis of high-nuclearity silver clusters? Furthermore, what effects does this have on the formation of the ultimate silver clusters?

Herein, we attempt to answer these unsolved puzzles and present the effect of different sodium phosphate salts on the formation of high-nuclearity silver clusters. An unprecedented high-nuclearity silver cluster, **Ag104a**, exhibiting a special silver-containing (PO_4_)@Ag_4_@(PO_4_)_12_ template layer and an outer Ag_100_ shell protected by *c*PrC≡C^−^ (*c*Pr = cyclopropyl) and Ph_2_PS_2_^−^ ligands, was isolated by introducing Na_3_PO_4_ or Na_2_HPO_4_ as the PO_4_^3−^ source in the presence of a base. To our knowledge, **Ag104a** represents the silver alkynyl cluster with the maximum count tetrahedral oxyanions to date. Notably, we obtained the other new co-crystallized silver cluster **Ag104b·Ag108a** with different structures from **Ag104a** by controlling the release rate of PO_4_^3−^ using NaH_2_PO_4_. As revealed by the time-dependent ^31^P nuclear magnetic resonance (NMR), the release rates of PO_4_^3−^ anions play a crucial role in regulating their structures. These findings underscore the effectiveness of various forms of phosphates in achieving precise control over the assembly processes of high-nuclearity silver clusters, thereby advancing our understanding of the structural dynamics of silver clusters.

## RESULTS

The synthetic routes for **Ag104a** and **Ag104b·Ag108a** are summarized in Scheme 1b by using a facile one-pot solvothermal method. Briefly, **Ag104a** was synthesized by the reaction of [*c*PrC≡CAg]*_n_*, Na_3_PO_4_·12H_2_O, Ph_2_PS_2_·HEt_3_N, CF_3_SO_3_Ag and *N,N,N*',*N*'-tetramethylethylenediamine (TMEDA) in CH_3_OH at 70°C. Light-yellow block crystals of **Ag104a** were obtained by evaporation of the filtrate at room temperature for 2 d. **Ag104a** was first characterized by using single-crystal X-ray diffraction (SCXRD) and the SQUEEZE protocol in PLATON was employed to remove the electron contribution of highly disordered counter ions and solvents. A total of 1951 electrons were removed from the unit cell (*Z* = 2) (Fig. S1), which can be approximately assigned to five CF_3_SO_3_^−^, thirty-three CH_3_OH and one H_2_O per formula. Therefore, the total formula of **Ag104a** is {[Ag_104_(PO_4_)_13_(*c*PrC≡C)_48_(Ph_2_PS_2_)_12_]·5CF_3_SO_3_·33CH_3_OH·H_2_O}, which is further verified by using thermogravimetric analysis (Fig. S2) and electrospray ionization mass spectrometry (ESI-MS) hereinafter. Remarkably, the use of equimolar Na_2_HPO_4_·12H_2_O instead of Na_3_PO_4_·12H_2_O also led to the isolation of **Ag104a**. However, by exclusively replacing Na_3_PO_4_·12H_2_O with an equimolar amount of NaH_2_PO_4_·2H_2_O while maintaining the other conditions, we found that colorless crystals were formed (Scheme 1b and Fig. S3). SCXRD analysis revealed that it was a co-crystal (**Ag104b·Ag108a**) composed of two silver clusters: [(PO_4_)@Ag_4_@(PO_4_)_12_@Ag_100_S_4_(*c*PrC≡C)_40_(Ph_2_PS_2_)_16_]^+^ (**Ag104b**) and [(PO_4_)@Ag_8_@(PO_4_)_12_@Ag_100_S_4_(*c*PrC≡C)_40_(Ph_2_PS_2_)_16_]^5+^ (**Ag108a**) at a ratio of 1:1 (Fig. S4) with partial site-occupancy disordered Ag_4_ and Ag_8_ cores, respectively. The co-crystallized silver nanoclusters have sporadically been observed in paired Ag_40_/Ag_46_ [53] and Ag_210_/Ag_211_ [20] with and without site-occupancy disordered features, respectively. The S^2−^ should have originated from the P–S bond cleavage of Ph_2_PS_2_^−^ ligands [54,55]. Using KH_2_AsO_4_·2H_2_O, similar co-crystal **(Ag104c·Ag108b)** containing [(AsO_4_)@Ag_4_@(AsO_4_)_12_@Ag_100_S_4_(*c*PrC≡C)_40_(Ph_2_PS_2_)_16_]^+^ (**Ag104c)** and [(AsO_4_)@Ag_8_@(AsO_4_)_12_@Ag_100_S_4_(*c*PrC≡C)_40_(Ph_2_PS_2_)_16_]^5+^ (**Ag108b)** can also be obtained (Figs S3 and S5), indicating that the co-crystallization of large silver clusters in a single crystal is not accidental in this system. More detailed synthetic procedures and characterizations (Figs S6 and S7) are given in the Supporting Information.

### X-ray structures

SCXRD analysis reveals that **Ag104a** crystallized in a tetragonal *P*4_2_/*nmc* space group with *D*_4d_ symmetry. Its asymmetric unit contains a quarter of the Ag_104_ cluster and the entirety exhibits a multilayered rosette-shaped structure. As portrayed in Fig. S8, the approximate dimension of the whole Ag_104_ cluster is 3.0 nm × 3.0 nm × 2.3 nm and its metallic skeleton dimension is 1.9 nm × 1.9 nm × 1.1 nm. The Ag_104_ cluster comprises 104 silver atoms, 13 PO_4_^3−^ and 60 organic ligands, including 48 *c*PrC≡C^−^ and 12 Ph_2_PS_2_^−^. The presence of the CF_3_SO_3_^−^ counterion, although unresolved crystallographically, has been validated by using ESI-MS (Fig. S9). The composition and purity of **Ag104a** were confirmed by using ^1^H NMR and ^31^P NMR (Figs S10 and S11). The ^1^H NMR displays two sets of peaks at 0–2 and 7–9 ppm, corresponding to the H atoms of *c*PrC≡C^−^ and Ph_2_PS_2_^−^, respectively. The peaks centered at 65–70 and 15–22 ppm in ^31^P NMR are assigned to the P atoms of Ph_2_PS_2_^−^ and PO_4_^3−^, respectively, which indicates dynamic averaging among chemically equivalent P atoms, as shown in the solid-state structure of **Ag104a**.

**Figure 1.**
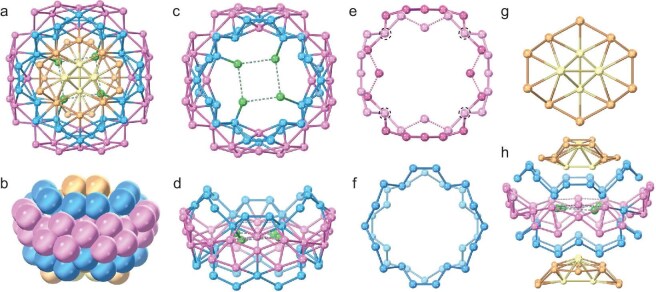
Structure dissection of silver skeleton in **Ag104a**. (a) Top view of the overall Ag_104_ core. (b) Front view of the overall Ag_104_ core in a space-filling mode. (c) Top and (d) front views of Ag_76_ consisting of a distorted Ag_4_ square encircled by an Ag_72_ garland. (e) Ag_36_ shell interlaced together by two Ag_20_ rings sharing four intersections highlighted by black dashed circles. (f) Two smaller Ag_18_ rings. (g) Two Ag_14_ units containing Ag_4_ tetrahedron at the poles of the whole silver skeleton (h). Dashed lines indicate Ag···Ag distances of >3.44 Å corresponding to the absence of argentophilic interaction [56]. All atoms with different colors are silver atoms.

**Figure 2.**
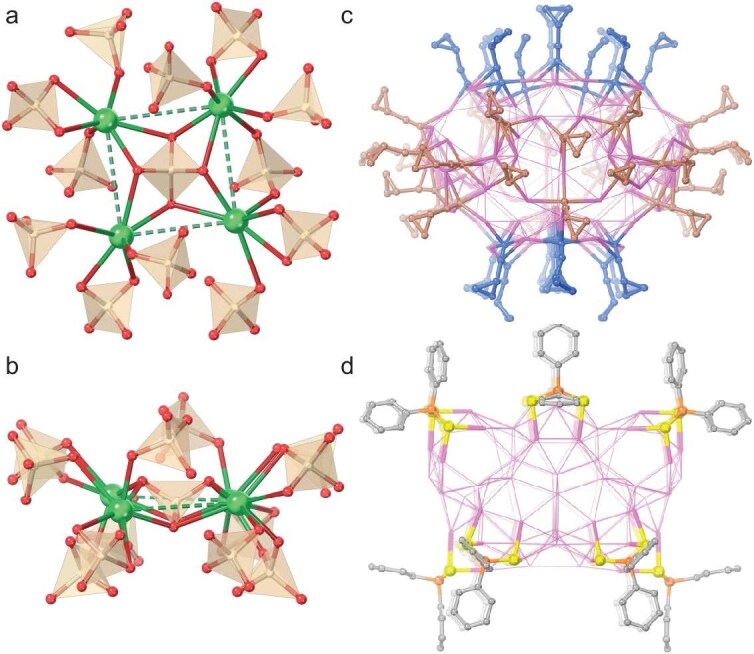
(a) Top and (b) front views of the silver-containing (PO_4_)@Ag_4_@(PO_4_)_12_ template layer in **Ag104a**. Dashed lines indicate Ag···Ag distances of >3.44 Å. PO_4_^3−^ anions are highlighted as pale yellow tetrahedrons. (c) Distribution of *c*PrC≡C^−^ ligands at the equator (brown) and poles (dark blue) on the Ag_100_ shell of **Ag104a**. (d) Distribution of 12 Ph_2_PS_2_^−^ ligands on the Ag_100_ shell of **Ag104a**. Color legend: Ag, green and pink; P, orange; S, yellow; O, red; C, dark blue, brown and gray.

A more detailed anatomy was performed to gain in-depth insight into its structure (Fig. 1). The entire silver skeleton of the Ag_104_ cluster can be divided into three hierarchies. In the center of the cluster is a distorted Ag_4_ square without argentophilic interaction, which is encircled by a garland-shaped Ag_72_ shell (Fig. 1c and d). The Ag_72_ garland is intricately woven by two Ag_20_ rings sharing four intersections to form Ag_36_ rings (Fig. 1e), further reinforced by two smaller Ag_18_ rings up and down (Fig. 1f). Positioned both above and below Ag_72_ is an Ag_14_ unit housing a central Ag_4_ tetrahedron within its confines (Fig. 1g and h, and Fig. S12). Notably, this Ag_4_ tetrahedron was known as the basic building unit commonly appearing in the silver cluster [57–61], but an example of such an appearance on the surface of a cluster has been rarely found [60,61]. Alternatively, it also can be seen as a distorted Ag_4_ square wrapped in an integrated Ag_100_ shell (Fig. S13). The argentophilic interactions (Ag···Ag) fall in the range of 2.75–3.37 Å and contribute to the stability of the overall metallic framework. Within the Ag_104_ cluster, there are a total of 13 PO_4_^3−^ anions supporting the silver skeleton through Ag–O bonds and they are symmetrically distributed around the crystallographic 4-fold rotoinversion axis. The innermost PO_4_^3−^ anion is fixed in the center of the distorted Ag_4_ square in a *μ*_8_-*κ*^2^:*κ*^2^:*κ*^2^:*κ*^2^ mode (Fig. S14), which is represented as a centered four-pointed star [(PO_4_)@Ag_4_]. Each silver atom on the distorted Ag_4_ square ligates with three or four additional PO_4_^3−^ anions to form the secondary anionic unity to support the outmost silver shell (Fig. 2a and b). These 12 PO_4_^3−^ anions adopt *μ*_11_-*κ*^2^:*κ*^3^:*κ*^3^:*κ*^3^ and *μ*_12_-*κ*^2^:*κ*^3^:*κ*^3^:*κ*^4^ modes (Ag–O: 2.13–2.80 Å) at a ratio of 2:1. Specifically, the PO_4_^3−^ anion plays three roles in the construction of **Ag104a**: (i) passivating the Ag_4_ core; (ii) supporting the Ag_100_ shell; and (iii) connecting the core and shell. It is noticed that the outermost *c*PrC≡C^−^ and Ph_2_PS_2_^−^ ligands regioselectively cover the Ag_100_ shell of the Ag_104_ cluster. The 48 *c*PrC≡C^−^ can be divided into two layers: 10 *c*PrC≡C^−^ ligands adopt *μ*_4_-*η*^1^:*η*^1^:*η*^1^:*η*^1^, *μ*_3_-*η*^1^:*η*^1^:*η*^1^ and *μ*_2_-*η*^1^:*η*^1^ coordination patterns at a ratio of 3:1:1, capping at the opposite two poles of the silver skeleton, and the remaining 28 are distributed near to the equator via 12 *μ*_3_-*η*^1^:*η*^1^:*η*^1^, 12 *μ*_4_-*η*^1^:*η*^1^:*η*^1^:*η*^1^ and 4 *μ*_4_-*η*^1^:*η*^1^:*η*^1^:*η*^2^ coordination patterns (Fig. 2c and Fig. S15) (Ag–C bond lengths: 2.04–2.68 Å). A total of 12 bidentate Ph_2_PS_2_^−^ ligands, 6 in one group, are inserted between the *c*PrC≡C^−^ layers (Fig. 2d and Fig. S16). Each caps on the square face in a *μ*_4_-*η*^2^:*η*^2^ binding mode and the Ag–S bond lengths fall within the range of 2.53–2.59 Å.

**Figure 3.**
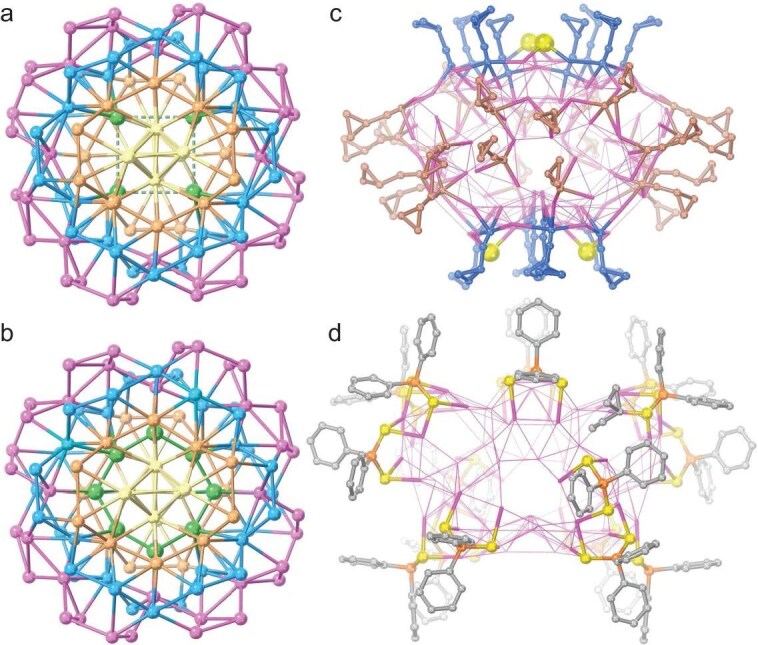
(a) Overall Ag_104_ framework in **Ag104b**. (b) Overall Ag_108_ framework in **Ag108a**. (c) Distribution of *c*PrC≡C^−^ ligands at the equator (brown) and poles (dark blue) on the Ag_100_ shell of both Ag_104_ and Ag_108_ clusters in **Ag104a·Ag108a**. (d) Distribution of 16 Ph_2_PS_2_^−^ ligands on the Ag_100_ shell of **Ag104a·Ag108a**. Dashed lines indicate Ag···Ag distances of >3.44 Å. Color legend: Ag, pink, blue, apricot, buff and green; P, orange; S, yellow; C, dark blue, brown and gray.

**Ag104b·Ag108a**, a mixture of Ag_104_ and Ag_108_ at a 1:1 ratio, are crystalized in a tetragonal *I*${\bar{4}} $ space group. As in **Ag104a**, the Ag_104_ cluster in **Ag104b·Ag108a** also has an Ag_4_ square, connected with 13 PO_4_^3−^, forming a silver-containing (PO_4_)@Ag_4_@(PO_4_)_12_ template layer (Figs S17 and S18). In contrast, the Ag_108_ cluster displays a folded Ag_8_ octagon with a uniform Ag···Ag distance of 2.66 Å, indicating the presence of nontrivial argentophilic interactions. Housing in the Ag_8_ unit is one PO_4_^3−^ tetrahedron in a *μ*_12_-*κ*^3^:*κ*^3^:*κ*^3^:*κ*^3^ coordination mode, represented as [(PO_4_)@Ag_8_] (Figs S17–S19), which is further linked with 12 PO_4_^3−^, forming a silver-containing (PO_4_)@Ag_8_@(PO_4_)_12_ template layer. Both silver-containing template layers are encapsulated by the same Ag_100_ shell (Fig. 3a and b), which can also be viewed as an Ag_72_ garland capped by two Ag_14_ units (Fig. S18). Moreover, Ag_104_ and Ag_108_ clusters have identical ligand layers composed of 40 *c*PrC≡C^−^, 16 Ph_2_PS_2_^−^ and 4 S^2−^, with the same total count of ligands as **Ag104a** (48 *c*PrC≡C^−^ and 12 Ph_2_PS_2_^−^). Notably, unlike in **Ag104a**, both Ag_104_ and Ag_108_ clusters feature 8 *c*PrC≡C^−^ and 2 *μ*_3_-bridging S^2−^ ligands at the opposite two poles of the silver skeleton, of which two S^2−^ ligands occupy the two *c*PrC≡C^−^ positions in **Ag104a** (Ag–S bond lengths: 2.33–2.62 Å), which leads to lower *S*_4_ symmetry, leaving 24 *c*PrC≡C^−^ ligands located at the equator (Figs 2c, 3c and Fig. S20). The Ag–C distances are in the range of 1.99–2.68 Å. Except for the 12 *μ*_4_-mode Ph_2_PS_2_^−^ inserted between the *c*PrC≡C^−^ layers (Fig. 3d and Fig. S21), there are still 4 *μ*_4_-mode Ph_2_PS_2_^−^ cooperating with the equatorial 24 *c*PrC≡C^−^ to stabilize the silver shell (Ag–S bond lengths: 2.49–2.62 Å). Furthermore, the composition of **Ag104b·Ag108a** was confirmed by using ^1^H NMR and ^31^P NMR (Figs S10 and S11). The ^31^P NMR displays a set of peaks centered at ∼20 ppm, evidencing the presence of only PO_4_^3−^ anions in **Ag104b·Ag108a**, without HPO_4_^2−^ and H_2_PO_4_^−^ anions. Additionally, four peaks at 67–71 ppm at a ratio of 1:1:1:1 are assigned to the P atoms of Ph_2_PS_2_^−^ in four different chemical environments, as shown in the structure of the ligand layer of both Ag_104_ and Ag_108_ clusters in **Ag104b·Ag108a**.

### Phosphate balance-regulating assembly of silver clusters

As observed in Ag_104_ and Ag_108_ clusters from **Ag104b·Ag108a**, only trivalent tetrahedral PO_4_^3−^ anion was ultimately found as a template, despite the H_2_PO_4_^−^ being used in the synthesis of the silver clusters. One potential factor contributing to the structural difference in **Ag104a** and **Ag104b·Ag108a** may be attributed to variances in the release rate of PO_4_^3−^ in solution. Under a weakly alkaline environment created by TMEDA, Na_3_PO_4_ directly released PO_4_^3−^ whereas the H_2_PO_4_^−^ of the NaH_2_PO_4_ slowly transformed into PO_4_^3−^. TMEDA can consume H^+^ ions as in the following equation: (CH_3_)_2_N(CH_2_)_2_N(CH_3_)_2_ + 2H^+^ → [(CH_3_)_2_NH(CH_2_)_2_NH(CH_3_)_2_]^2+^ [62], which promotes the equilibrium of H_2_PO_4_^−^ toward the release of PO_4_^3−^ anions (H_2_PO_4_^−^ ⇆ H^+^ + HPO_4_^2−^, HPO_4_^2−^ ⇆ H^+^ + PO_4_^3−^) [63]. In a previous example, we observed three phosphate species (H_2_PO_4_^−^, HPO_4_^2−^ and PO_4_^3−^) coexisting in a thiolate-protected Ag_102_ cluster when H_2_PO_4_^−^ ion was utilized without a base in the reaction system [47]. This also emphasizes the essential role of TMEDA in the formation of silver clusters in this system, as it provides a weak base environment to control the slow release of PO_4_^3−^ from H_2_PO_4_^−^ (Table S1).

**Figure 4.**
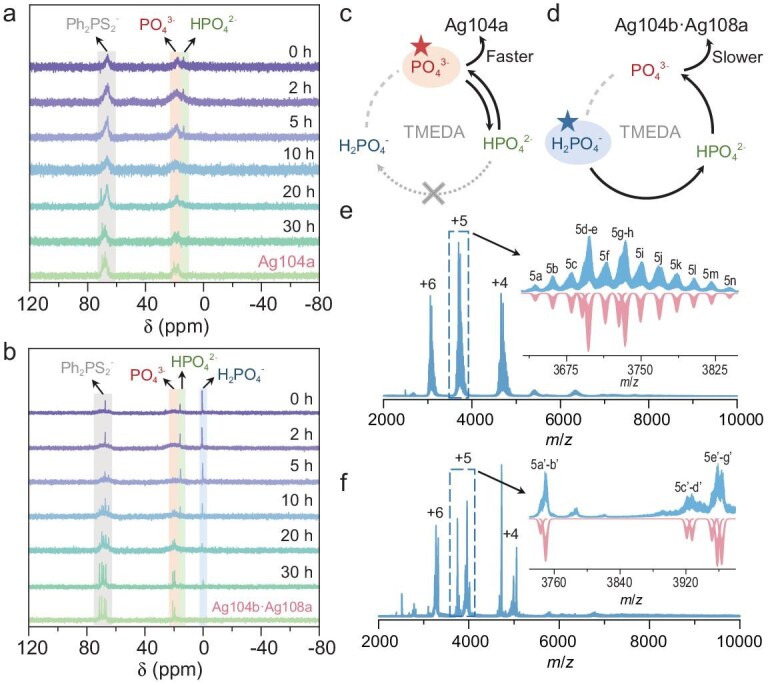
Time-dependent ^31^P NMR of reaction solution for (a) **Ag104a** and (b) **Ag104b·Ag108a**. Schematic representation of the dissociation equilibrium of [H_3−_*_x_*PO_4_]*^x^*^−^ in the reaction solution of (c) **Ag104a** and (d) **Ag104b·Ag108a**. Stars indicate starting materials. Positive-ion mode ESI-MS of (e) **Ag104a** and (f) **Ag104b·Ag108a**. Inset: Zoom-in mass spectra of experimental (blue line) and simulated (pink line) isotope patterns for +5 labeled species.

^31^P NMR has proven to be an effective method for distinguishing various [H_3−_*_x_*PO_4_]*^x^*^−^ species that are present in the reaction mixture during cluster synthesis [47]. Thus, to further verify the above deduction, time-dependent ^31^P NMR was used to track the synthesis of **Ag104a** and **Ag104b·Ag108a**. As anticipated, the ^31^P NMR analysis of **Ag104a** revealed a characteristic signal of PO_4_^3−^ that appeared at 18.43 ppm, even at the initial reaction stage, concurrently with HPO_4_^2−^ (13.8 ppm, from the partial hydrolysis of PO_4_^3−^) and a broad Ph_2_PS_2_^−^ envelope (65–70 ppm, centered at 66 ppm, reflecting the formation of Ag–S bonds) (Fig. 4a). Notably, these chemical shifts show significant downfield displacements compared with reference sodium phosphate salt [47], indicating the occurrence of phosphate-templated nucleation. With progression, the HPO_4_^2−^ signal diminished entirely, leaving the single signal of PO_4_^3−^. The ^31^P NMR of the reaction solution after 30 h exhibits nearly identical signals to those of **Ag104a** dissolved in CD_2_Cl_2_, indicating the formation of **Ag104a**. In contrast, in the ^31^P NMR of **Ag104b·Ag108a** before solvothermal treatment, only signals for H_2_PO_4_^−^ and HPO_4_^2−^ were clearly detected at 0.64 and 15.87 ppm, respectively (Fig. 4b). Critical spectral transitions occurred at 10 h: emergent PO_4_^3−^ anions (64–71 ppm) mark template deprotonation and core fixation, while the Ph_2_PS_2_^−^ envelope evolves from a single peak (67.33 ppm) to split into multiple peaks (67–71 ppm), indicating coordination symmetry at silver cluster interfaces. Furthermore, the ^31^P NMR of the reaction solution after reacting for 30 h exhibits almost identical signals to those of **Ag104b·Ag108a** dissolved in CD_2_Cl_2_, suggesting their formation in the solution. These results indicate that the release rate of PO_4_^3−^ in **Ag104b·Ag108a** is slower than that in **Ag104a**. This is because H_2_PO_4_^−^ undergoes two chemical reactions involving deprotonation to release PO_4_^3−^ in a weakly alkaline environment (Fig. 4c and d) whereas Na_3_PO_4_ directly releases PO_4_^3−^.

### ESI-MS of **Ag104a** and **Ag104b·Ag108a**

The ESI-MS technique was further employed to verify the compositions of these silver clusters. As shown in Fig. 4e, the ESI-MS of **Ag104a** dissolved in CH_2_Cl_2_ in the positive-ion mode shows three major groups of peaks, each corresponding to different charge states of species: +6 species (*m/z* = 3000–3170), +5 species (*m/z* = 3630–3850) and +4 species (*m/z* = 4580–4820). The most prominent set of peaks is a group of signals consisting of 14 +5 species (**5a**–**5n**), of which **5f** at the *m/z* = 3713.92 is assigned to [Ag_104_(PO_4_)_13_(*c*PrC≡C)_48_(Ph_2_PS_2_)_12_]^5+^ (calcd. *m/z* = 3713.87), which can be regarded as the molecular ion of **Ag104a. 5g**–**5n** contain an intact Ag_104_ cluster appended by solvents or ions, whereas **5a**–**5e** are formed by ligand exchange in the Ag_104_ cluster (Fig. S22). In the other two envelopes, all of the +4 (**4a**–**4 m**) and +6 (**6a**–**6l**) species also contain a complete 104-nuclei silver framework, as evidenced by the assigned formula by matching experimental and simulated isotopic distributions (Figs S23 and S24). From the small-angle X-ray scattering results, **Ag104a** dispersed in solution is relatively stable without aggregation (Fig. S25) and it can be modeled as a sphere (Fig. S26) with dimensions that are close to those for the nanocluster obtained from crystal data [64–66].

Moreover, ESI-MS provides powerful evidence to verify the coexistence of two different silver clusters in **Ag104b·Ag108a** [53]. To guarantee the purity of the sample, the single crystals were selected to perform measurement in CH_2_Cl_2_–CH_3_OH mixed solvents. Similarly to **Ag104a**, there are mainly also three grouped peaks with +6, +5 and +4 species, but the groups of +5 and +4 species show two relatively separated peaks rather than the consecutive peaks that were observed in **Ag104a** (Fig. 4f). Among them, **5b'** at *m/z* = 3949.27 is assigned to [Ag_104_(PO_4_)_13_S_4_(*c*PrC≡C)_37_(Ph_2_PS_2_)_15_(H_2_O)]^5+^ (calcd. *m/z* = 3749.36), which can be attributed to the molecular ion of the Ag_104_ cluster in **Ag104b·Ag108a** by removing three *c*PrC≡C^−^ ligands and one Ph_2_PS_2_^−^ ligand but adding one H_2_O molecule (Fig. S27). Of note, **5c'** centered at *m/z* = 3921.02 corresponds to [Ag_108_(PO_4_)_13_S_4_(*c*PrC≡C)_40_(Ph_2_PS_2_)_16_]^5+^ (calcd. *m/z* = 3921.10), which can be attributed to the molecular ion of the Ag_108_ cluster in **Ag104b·Ag108a** (Fig. S27). Similarly, the compositions of both the Ag_104_ cluster and the Ag_108_ cluster can also be detected in the set of +4 species (Fig. S28). Comparison of the experimental (pink line) and simulated (blue line) isotopic distributions confirms the formula assignments. By contrast, only the species of the Ag_108_ cluster can be identified in the set of peaks with +6 valence (Fig. S29). The above observations prove the real presence of two clusters in a single crystal of **Ag104b·Ag108a**.

### Theoretical analysis of **Ag104a**

**Figure 5.**
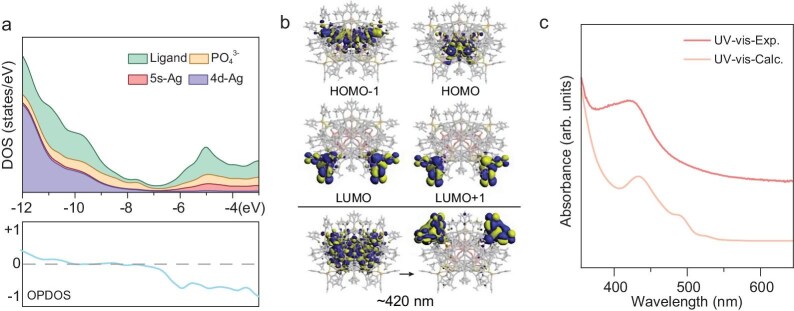
(a) Density of states for **Ag104a**, denoting the contribution from 4d-Ag, 5s-Ag, PO_4_^3−^ and ligand-based levels and the respective Ag-PO_4_^3−^ overlap denoting bonding (positive) and anti-bonding (negative) contributions. (b) Frontier orbitals denoting the highest occupied and lowest unoccupied molecular orbitals (HOMO and LUMO) and the orbitals involved in the main UV–vis absorbance are given for **Ag104a**. (c) Experimental and calculated UV–vis absorbance profiles for **Ag104a**.

In order to gain further insights into the electronic structure of the ligand-protected silver phosphate cluster, computational calculations on **Ag104a** were carried out. The relaxed geometries of the metal backbone are in good agreement with the experimentally characterized results, as denoted by the root-mean-square deviation (RMSD) of 0.099 Å for **Ag104a** in comparison with the X-ray structures. For the ligand-protecting layer provided by external organic ligands, the RMSD increases to 0.475 Å, denoting a deviation from the experiment and theory, owing to the fact that such a layer is more affected by packing effects in the crystal structure. The density of states is able to expose the contribution of the electronic shells in terms of the constitutive protecting-layer-, phosphate- and 4d/5s-Ag-based orbitals (Fig. 5a). The lower occupied levels are dominated by 4d-Ag/PO_4_/ligand contributions, turning to a main PO_4_^3−^ character at the high-lying occupied orbitals, denoting a ligand character for the low-lying unoccupied orbitals, with contributions of the 5s-Ag levels (Fig. 5b), resulting in a calculated highest occupied molecular orbital (HOMO)–lowest unoccupied molecular orbital (LUMO) gap of 1.00 eV for the overall Ag_104_ cluster. The appearance of 5s-Ag levels as part of the unoccupied molecular orbitals manifold denotes that the metal centers are formally Ag(I) ions, as supported by the Hirshfeld charge analysis averaging to +0.65 ē per Ag atom within the Ag_104_ core backbone. The obtained ultraviolet-visible (UV-vis) spectrum for **Ag104a** exhibits a distinctive peak at ∼420 nm. The simulated UV–vis pattern shows a shoulder at 435 nm (Fig. 5c). The ∼420-nm peak in **Ag104a** is of the main PO_4_/4d-Ag-to-ligand charge transfer, centered at the aryl rings at Ph_2_PS_2_^−^ moieties, thus ascribed as a core-to-ligand character [67].

Furthermore, in order to evaluate the role of the encapsulated PO_4_^3−^ anions, an energy decomposition analysis (EDA) [68] of the interaction energy between PO_4_^3−^ and [Ag_4_@(PO_4_)_12_Ag_100_(*c*PrC≡C)_48_(Ph_2_PS_2_)_12_]^8+^ was conducted for **Ag104a**. The calculated interaction energy (Δ*E*_int_) amounts to −1705.1 kcal/mol, denoting that the encapsulation of PO_4_^3−^ anions is largely favored. The EDA analysis is able to decompose the interaction energy into chemically meaningful terms accounting for the electrostatic (Δ*E*_elstat_), orbital (Δ*E*_orb_) and London dispersion (Δ*E*_disp_), which are stabilizing contributions accounting for the efficient PO_4_^3−^ encapsulation. Such values denote a stabilizing contribution of 55.2% from the electrostatic term, which amounts to −1304.4 kcal/mol for the formation of **Ag104a**, and of 43.7% from the orbital term, amounting to −1033.2 kcal/mol for the cluster. In addition, a small contribution from London dispersion is given, accounting for 1.1% of the stabilizing interactions, amounting to −25.6 kcal/mol. Thus, efficient encapsulation of PO_4_^3−^ is given mainly by both the electrostatic and the orbital character of the interaction.

Moreover, we computationally explore both the structural and the electronic changes upon removal of the central PO_4_^3−^ anion in order to evaluate its role in the resulting characteristics of the overall Ag_104_ cluster. Geometry relaxation of the hypothetical hollow [Ag_4_@(PO_4_)_12_Ag_100_(*c*PrC≡C)_48_(Ph_2_PS_2_)_12_]^8+^ counterpart shows an increased distortion of the metallic backbone of the Ag_104_ cluster, as given by the RMSD values of 0.269 Å in comparison with the parent cluster. Hence, the PO_4_^3−^ ion is relevant to retain the Ag_104_ core architecture within the cluster. The removal of the central PO_4_^3−^ ion leads to a decrease in the HOMO–LUMO gap to 0.64 eV, which leads to the strong variation in the UV–vis absorption profile (Figs S30 and S31). The charge transfer is estimated via the Hirshfeld charge analysis leading to a charge of −0.06 e for the central PO_4_^3−^ unit, denoting that the orbital interaction term from the EDA is given by the sizable PO_4_^3−^→[Ag_4_@(PO_4_)_12_Ag_100_(*c*PrC≡C)_48_(Ph_2_PS_2_)_12_]^8+^ charge transfer of 2.94 e. Thus, the central PO_4_^3−^ unit serving as a structural template and modifier of the both frontier orbital and UV–vis features for the overall **Ag104a** suggests the term ‘non-innocent templates’, which was coined to denote embedded ions or molecular motifs that were able to tune the inherent cluster characteristics.

### Optical properties of **Ag104a**

The solid-state UV–vis absorption spectrum of **Ag104a** was measured at room temperature. The cluster exhibits broad absorption across the UV and visible regions, with absorption in the range of 330–495 nm (Fig. S32a). The band gap of **Ag104a** was determined as 1.26 eV by using the Kubelka–Munk and Tauc functions, which indicates the narrow gap semiconductor nature (Fig. S32b). **Ag104a** exhibits a weak photoluminescence (PL) band near the edge of the visible region with a peak centered at 616 nm under an excitation of 480 nm, producing a large Stocks shift (i.e. 1239.83/480 = 2.58 eV to 1239.83/616 = 2.01 eV). The PL dynamics were studied by using the time-correlated single-photon counting technique; two microsecond components at 83 K (*τ*_1_ = 0.97 μs, *B*_1_ = 44.29%, *τ*_2_ = 12.18 μs, *B*_2_ = 55.71%) are required to fit the decay for **Ag104a**, giving rise to an average lifetime of 11.54 μs. In the same way, two lifetime components for **Ag104a** at 293 K (*τ*_1_ = 0.58 μs, *B*_1_ = 6.25%, *τ*_2_ = 11.58 μs, *B*_2_ = 93.75%) are required, with an average lifetime of 11.51 μs. The fitting results suggest that the PL may originate from a triplet excited state with two relaxation channels [69]. To verify the triplet characteristic, electron paramagnetic resonance spectra were measured to confirm the singlet oxygen (^1^O_2_) generation by using 2,2,6,6-tetramethylpiperidine (TEMP) as the trapping reagent. **Ag104a** shows a significant ^1^O_2_ signal under xenon-lamp irradiation (Fig. S33) in the solid state, suggesting its triplet spin-multiplicity of the excited state.

**Figure 6.**
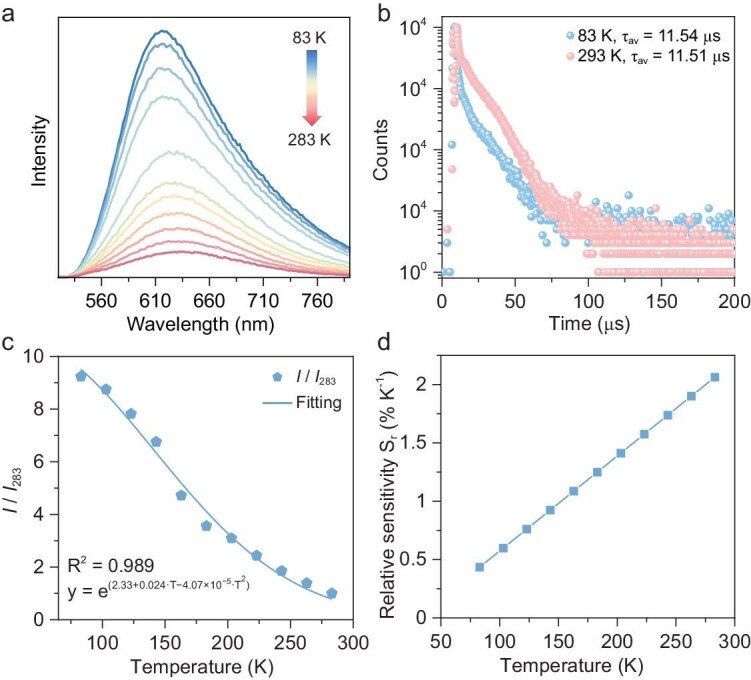
(a) Temperature-dependent PL spectra of **Ag104a** in the solid state from 83 to 283 K with 20 K as an interval. (b) PL decay traces of **Ag104a** at 83 and 293 K. (c) Normalized integrated peak intensities and fitting by using Equation (1) (data from (a)). (d) Relative thermal sensitivity *S*_r_ as a function of temperature.

To study temperature-dependent emission behaviors, the emission spectra (*λ*_ex_ = 480 nm) of **Ag104a** in the solid state were collected from 83 to 283 K, with 20 K as an interval. As presented in Fig. 6a, the PL intensity exhibits a negative correlation with temperatures from 283 to 83 K, in which the emission intensity at 83 K showcases a nearly 9.5-fold increase compared with that at 283 K. Moreover, the PL position gradually shifted from 636 to 616 nm during the cooling process, which corresponds to an increase in the PL energy level. Meanwhile, the PL lifetimes of **Ag104a** at 283 and 83 K were almost unchanged, with both being ∼11.5 μs (Fig. 6b), reflecting that the relaxation channels and lifetimes of excited electrons are temperature-independent. As is widely reported, the temperature-dependent emissions should be in connection with the variations in molecule rigidity and argentophilic interactions [70–72]. Therefore, we tentatively deduced that the blue shift in the temperature-dependent PL of **Ag104a** should be attributed to the triplet core-to-ligand charge transfer (^3^MLCT) from the Ag atoms to the *c*PrC≡C^−^ and Ph_2_PS_2_^−^ligands and is more susceptible to temperature than the cluster-center (^3^CC)-dominated PL. As shown in Fig. 6c, with increasing temperature, the relative emission intensity of **Ag104a** (Δ = *I*/*I*_283_, *I*_283_ denotes the integrated intensity of the band at 283 K) decreases. The temperature-dependent emission intensity can be well fitted via Equation (1):


(1) \begin{eqnarray*}
\Delta = {{\mathrm{e}}}^{{\mathrm{(2}}{\mathrm{.33 + 0}}{\mathrm{.024}}T - 4.07 \times {{10}}^{ - 5} \cdot {T}^2)}
\end{eqnarray*}

with a correlation coefficient *R*^2^ of 0.989 for **Ag104**, which indicates that the cluster can be used as a luminescent thermometer between 83 and 283 K. The relative thermal sensitivity *S*_r_ (*S*_r_ = |$\partial $Δ/$\partial $T|/Δ) is used to characterize the performances of the thermometers. The *S*_r_ of **Ag104a** increases linearly with temperature, with a maximum value of 2.06% K^−1^ at 283 K (Fig. 6d). These good thermometric characteristics and a wide temperature measurement range for **Ag104a** make it potentially valuable for optical thermometer applications [73,74].

## CONCLUSION

In summary, we explore the release rate of PO_4_^3−^ in weakly alkaline environments by utilizing different forms of phosphates (orthophosphate, hydrogen phosphate and dihydrogen phosphate) as a means to regulate the assembly of high-nuclearity silver clusters. Direct employment of Na_3_PO_4_ led to a multilayered rosette-shaped silver cluster **Ag104a**, which possesses a unique silver-containing (PO_4_)@Ag_4_@(PO_4_)_12_ template layer. Conversely, when utilizing NaH_2_PO_4_ to indirectly release PO_4_^3−^, we obtained the other new co-crystallized silver cluster **Ag104b·Ag108a**. Furthermore, time-dependent ^31^P NMR was used to track the synthesis of **Ag104a** and **Ag104b·Ag108a** and the results revealed that different release rates of the PO_4_^3−^ anions can affect the assembly of silver clusters. This work not only demonstrates silver alkynyl clusters containing the maximum count of tetrahedral oxyanions, but also provides crucial insights into regulating the assembly of high-nuclearity silver clusters by phosphate dissociation balance and opportunities for researchers to understand the structural dynamics of silver clusters.

## Supplementary Material

nwaf183_Supplemental_Files

## Data Availability

The data that support the findings of this study are available within the article and its supplementary information files. Other relevant data are available from the corresponding author upon request. The X-ray crystallographic coordinates for structures reported in this article have been deposited at the Cambridge Crystallographic Data Centre, under deposition numbers CCDC 2410600, 2410601 and 2410602 for **Ag104a, Ag104b·Ag108a** and **Ag104c·Ag108b.** These data can be obtained free of charge from the Cambridge Crystallographic Data Centre via www.ccdc.cam.ac.uk/data_request/cif.
