# Supplementary material for: Bioinspired phosphate homeostasis for atomically precise regulation of silver nanoclusters
Source: Natl Sci Rev. 2025 May 10;12(7):nwaf183. doi: 10.1093/nsr/nwaf183 (PMC12205487; doi:10.1093/nsr/nwaf183)
Supplement: nwaf183_Supplemental_Files [file nwaf183_supplemental_files.zip › 2025-350-SI.pdf]

Supporting Information (SI)

Bioinspired Phosphate Homeostasis for Atomically Precise Regulation of Silver Nanoclusters

Wei-Dan Si,[†] Lu-Yang Xing,[†] Álvaro Muñoz-Castro,[‡] Chengkai Zhang,[†] Bao-Liang Han,[†] Jian-Long Zhou,[†] Zhi Wang,[*,†] Chen-Ho Tung,[†] and Di Sun[*,†]

[†]School of Chemistry and Chemical Engineering, State Key Laboratory of Crystal Materials, Shandong University, Ji'nan, 250100, P. R. China.
[‡]Facultad de Ingeniería, Arquitectura y Diseño, Universidad San Sebastián, Bellavista 7, Santiago, 8420524, Chile.

Materials and Instruments

[cPrC≡CAg]$_n$[1] and Ph_2PS_2·$HNEt_3$[2] were synthesized according to the literature. cPrC≡CH (Adamas-beta®), Ag_2O (Adamas-beta®), CF_3SO_3Ag (Adamas-beta®), and TMEDA (Adamas-beta®) were purchased from Shanghai Titan Scientific Co., Ltd. All other reagents employed were commercially available and used as received without further purification.

IR spectra were recorded on a Bruker Tensor II spectrophotometer (Bruker Optics GmbH, Ettlingen, Germany) utilizing a single attenuated total reflectance (ATR) accessory covering a wavenumber range from 400 to 4000 cm^{-1}. The final spectrum was the average of 32 scans accumulated using Bruker's Opus software 8.1, taken at 4 cm^{-1} resolution. The samples were measured under the same mechanical force pushing the samples in contact with the diamond window.

UV-vis absorbance spectra were recorded on a Thermo Scientific Evolution 220 UV-visible spectrophotometer. UV-vis diffuse reflectance spectra (DRS) were recorded on a Thermo Scientific Evolution 220 UV-visible spectrophotometer equipped with a 60 mm integrating sphere and SPECTRALON® was used as the reference.

Mass spectra (MS) were recorded on a Bruker impact II high definition mass spectrometer, quadrupole and time-of-flight (Q/TOF) modules. Typical measurement conditions are as follows: end plate offset = -400 V; dry gas = 3 L min^{-1}, nebulizer = 0.3 bar, capillary voltage = 3500 V, sample flow rate = 180 μL/h. The data analyses of mass spectra were performed based on the isotope distribution patterns using Compass Data Analysis software (Version 4.4).

Temperature-dependent photoluminescence measurements were carried out in an Edinburgh spectrofluorimeter (FLS920) coupled with an Optistat DN cryostat (Oxford Instruments), and the ITC temperature controller and a pressure gauge were used to realize the variable-temperature measurement in the range of 83-283 K. Spectra were collected at different temperatures after a 2 min homoiothermy. Time-resolved luminescence lifetime measurements were performed on the same instrument by using a time-correlated single-photon counting technique.

^{1}H NMR and ^{31}P NMR spectra were recorded by dissolving samples in CD_2Cl_2 at room temperature on a Bruker Avance 400.

Thermogravimetic analysis (TGA) was done in a TA SDT Q600 thermal analyzer at a heating

rate of 20 ℃/min under N_2 atmosphere (200 mL/min) from 20 to 820 ℃.

The Electron Paramagnetic Resonance (EPR) spectra were obtained with a JES-X320 EPR spectrometer (JEOL, Tokyo, Japan). The EPR spectra was analyzed with A-System EPR Date Processing Version 3.9.3.0 software.

SAXS measurements were performed using a SAXSess MC2 high flux SAXS instrument (Anton Paar, Austria, Cu Kα, λ = 0.154178 nm), equipped with a Kratky block-collimation system and using an image plate as the detector, reading the data from the image plate in a dark room. The concentration of sample in N,N'-Dimethylformamide (DMF) was 10 mg/mL and the SAXS pattern of pure DMF was collected as scattering background. Samples were inserted in a quartz capillary of inner diameter 1.0 mm. The sample-to-detector distances were 26.45 cm. The exposure time is 30 min.

X-ray Crystallography

Single crystals of **Ag104a**, **Ag104b·Ag108a**, and **Ag104c·Ag108b** with appropriate dimensions were selected under an optical microscope and rapidly coated with high vacuum grease (Dow Corning Corporation) to prevent decomposition. The crystal was mounted on a CryoLoop™ loop and the single-crystal X-ray diffraction (SCXRD) data of **Ag104a** was measured at 173 K. The intensity data and cell parameters of **Ag104a** was recorded on a Rigaku Oxford Diffraction XtaLAB Synergy diffractometer equipped with Rigaku Hypix detector and an Oxford Cryosystems Cryostream Plus 800 open-fow N_2 cooling device. A Mo Kα radiation (λ = 0.71073 Å) from PhotonJet micro-focus X-ray sources was used for the measurement. The diffraction images were processed using the *CrysAlis*^{Pro} software.

In addition, the crystals were mounted on the CryoLoop™ ring and single crystal X-ray diffraction (SCXRD) data for a crystal of **Ag104b·Ag108a** were measured at 153 K, and SCXRD data for a crystal of **Ag104c·Ag108b** at 173 K. The intensity data and cell parameters were recorded on Bruker D8 VENTURE diffractometer with an Incoatec IμS 3.0 Cu EF microfocus source (55W, Cu Kα, λ = 1.54178 Å) equipped with a PHOTON III C28 detector and an Oxford Cryosystems CryostreamPlus 800 open-fow N_2 cooling device. The raw frame data were processed using SAINT and SADABS to yield the reflection data file.

These structures were solved using the charge-flipping algorithm, as implemented in the program *SUPERFLIP*[3] and refined by full-matrix least-squares techniques against Fo^2 using the SHELXL program[4] through the OLEX2 interface.[5] Hydrogen atoms at carbon were placed in calculated positions and refined isotropically by using a riding model. Appropriate restraints or constraints were applied to the geometry and the atomic displacement parameters of the atoms in the cluster. All structures were examined using the Addsym subroutine of PLATON[6] to ensure that no additional symmetry could be applied to the models. Pertinent crystallographic data collection and refinement parameters are collated in Table S2-S4. Selected bond lengths and angles are collated in Table S5-S7.

Density functional theory (DFT) calculations.

The ADF code[7] incorporating scalar corrections via the ZORA Hamiltonian,[8] was employed for all the calculations. Double-ξ Slater basis set, plus one polarization function (STO-DZP) for valence electrons, were employed within the generalized gradient approximation (GGA) according to the Perdew-Burke-Ernzerhof (PBE) exchange-correlation functional.[9,10] Dispersion corrections to DFT were added via the pairwise Grimme3 approach.[11,12] The current level of theory (PBE/DZP) provides accurate results at an affordable computational cost with minimal ligand simplifications.[13,14] Optical properties were evaluated at the PBE/DZP level, where the calculated spectrum was blue-shifted by 0.30 eV, to overcome the expected theoretical error of the method of about ~0.15-0.30 eV, as denoted in previous calculations.[15,16] The frozen core approximation was applied to the [$1s^2$-$4p^6$] for Ag, [$1s^2$] for C and O, [$1s^2$-$2p^6$] for Cl, S, and P, leaving the remaining electrons to be treated variationally. Geometry optimizations were performed without any symmetry restrain via the analytical energy gradient method implemented by Versluis and Ziegler.[17] An energy convergence criterion of 10^{-4} Hartree, gradient convergence criteria of 10^{-3} Hartree/Å, and radial convergence criteria of 10^{-2} Å were employed for the evaluation of the relaxed structures.

Thermometric analysis

The competitive relationship between fluorescence intensity and radiation transition rate and non-radiation transition rate can be expressed as： $I(T) \propto \frac{\Gamma_{rad}}{\Gamma_{rad}+\Gamma_{nonrad}(T)}$; when the non-radiative transition dominant, this can be approximated as: $I(T) \propto e^{Ea/KT}$. However, in real materials, the non-radiative activation energy *Ea* is temperature-dependent due to the factors such as multi-phonon-assisted transition (acoustic and optical phonons) and lattice thermal expansion etc. To account for this, a quadratic polynomial correction is introduced: $\frac{Ea}{k} = a + bT + cT^2$; given that $\Delta T = \frac{\mathrm{I}(T)}{\mathrm{I}_{283}}$, it follows that $\Delta T = e^{a+bT+CT^2}$.

The *a* corresponds to the normalized constant at T_{ref}; the *b* as a linear term coefficient, describes the linear change of *Ea* with temperatures; *c* as the quadratic coefficient, represents the high order temperature effect.

Experimental details: the PL intensities I(*T*) of **Ag104a** were measured at different temperatures (83-283 K, with 20 K as an interval) under controlled temperature conditions, and the relative intensity ratios $\Delta(T)=I(T)/I_{283}$ were calculated using 283K as a reference, then the data were fitted using $\Delta T = e^{a+bT+CT^2}$.

Synthesis of Ag104a and Ag104b·Ag108a

To a CH_3OH (5 mL) solution containing [*c*PrC≡CAg]$_n$ (7 mg, 0.04 mmol), $Ph_2PS_2 \cdot HNEt_3$ (3.5 mg, 0.01 mmol), and CF_3SO_3Ag (12.8 mg, 0.05 mmol) in 100 μL N,N-Dimethylformamide (DMF), TMEDA (20 μL, 0.13 mmol) was added. After 10 min ultrasound (80W) at room temperature (20 ^{o}C), $Na_3PO_4 \cdot 12H_2O$ (7 mg, 0.02 mmol) was added into above mixed solution which was treated for further 10 min ultrasound under the same conditions. The mixture was sealed in a 25 mL Teflon-lined stainless-steel autoclave and kept at 70 ℃ for 30 h. After cooling, the orange solution was filtered and evaporated slowly in the dark at room temperature. **Ag104a** crystallized as faint yellow block crystals after 2-4 days with a yield of 1.7%. The synthesis of **Ag104b·Ag108a** was similar to **Ag104a**, except that $Na_3PO_4 \cdot 12H_2O$ was replaced by $NaH_2PO_4 \cdot 2H_2O$ (4 mg, 0.02 mmol), colorless crystals of **Ag104b·Ag108a** were crystallized for a week. Moreover, considering that TMEDA serves as a bidentate chelating ligand capable of coordinating with metal ions during the cluster synthesis, we conducted experiments substituting TMEDA with other bases (Et_3N and $NH_3 \cdot H_2O$) to rule out its potential chelating effect. As shown in Table S1, the successful synthesis of **Ag104a** under alkaline conditions (pH >8) using either Et_3N or $NH_3 \cdot H_2O$ in the presence of Na_3PO_4 demonstrates that the TMEDA's chelation capability is not essential for cluster formation. However, when same volume of Et_3N or $NH_3 \cdot H_2O$ was used in the synthesis of **Ag104b·Ag108a**, **Ag104a** was obtained due to the faster release of PO_4^{3-} anions. By further regulating the pH of reaction solution to ~7.7 through reduced usage of Et_3N or $NH_3 \cdot H_2O$, **Ag104b·Ag108a** was isolated, thereby confirming that no significant chelating effect from TMEDA was involved.

Synthesis of Ag104c·Ag108b

The synthesis of **Ag104c·Ag108b** was similar to **Ag104a**, except that $Na_3PO_4 \cdot 12H_2O$ was replaced by $KH_2AsO_4 \cdot 2H_2O$ (5 mg, 0.02 mmol), colorless crystals of **Ag104c·Ag108b** were crystallized for a week.

Figure S1: The crystal packing diagrams in one unit cell of Ag104a viewed along a (a), b (b) and c (c) axis. A unit cell contains 2 Ag104a. Color code: Ag, purple; P, orange; O, red; S, yellow; C, gray.

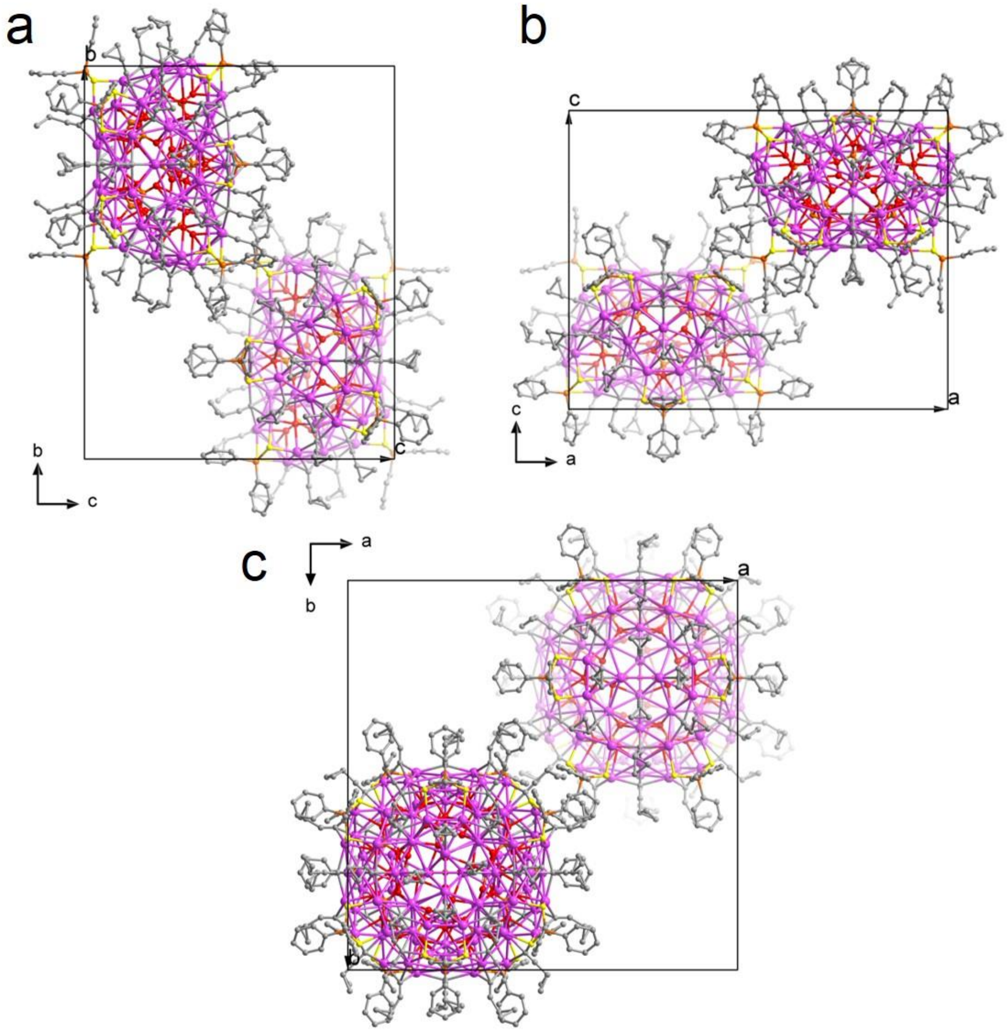

Figure S2: The thermogravimetric analysis of Ag104a.

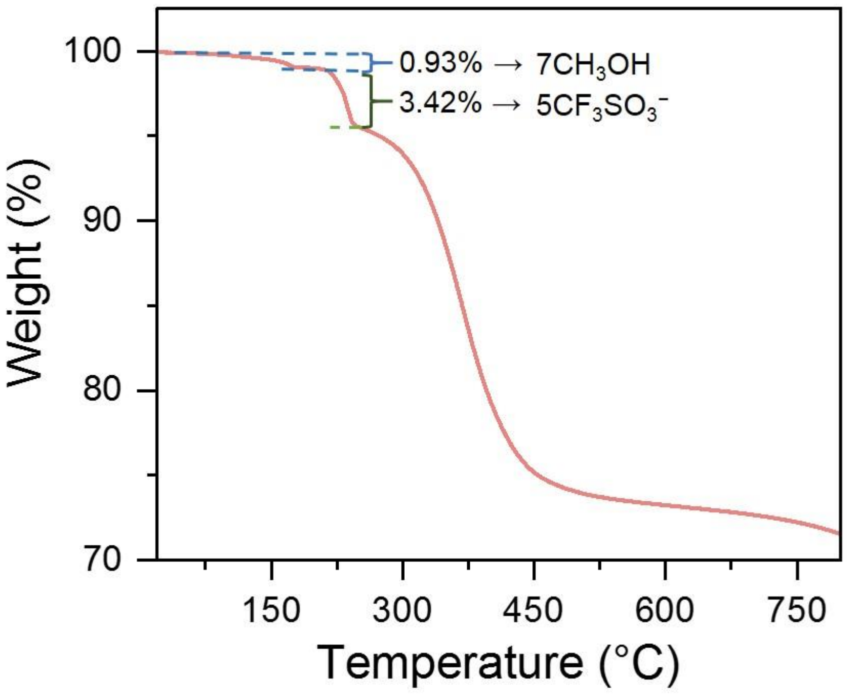

The TGA reveals that the mass loss during the temperature increase from 40 to 75 °C corresponds to the loss of 7 methanol in **Ag104a** (Calcd. 1.15%; Exp. 0.93%), and the mass loss between 75 and 245 °C corresponds to the loss of 5 $CF_3SO_3^-$ in **Ag104a** (Calcd. 3.84%; Exp. 3.42%).

Figure S3: The synthesis of these compounds is summarized.

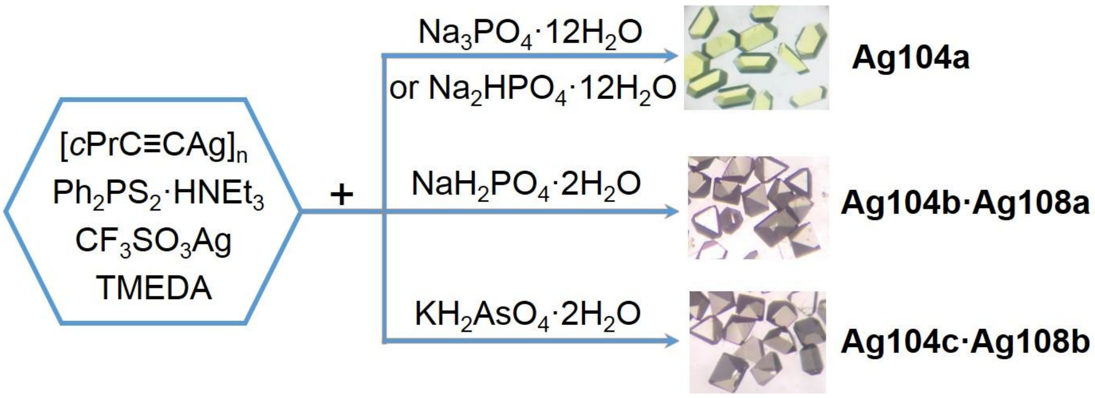

Figure S4: The top (a) and front (b) views of the total structure of Ag_{104} cluster in Ag104a·Ag108a. The top (c) and front (d) views of the total structure of Ag_{108} cluster in Ag104a·Ag108a. Hydrogen atoms are removed for clarity. Color legend: Ag, purple and green; P, orange; S, yellow; O, red; C, gray.

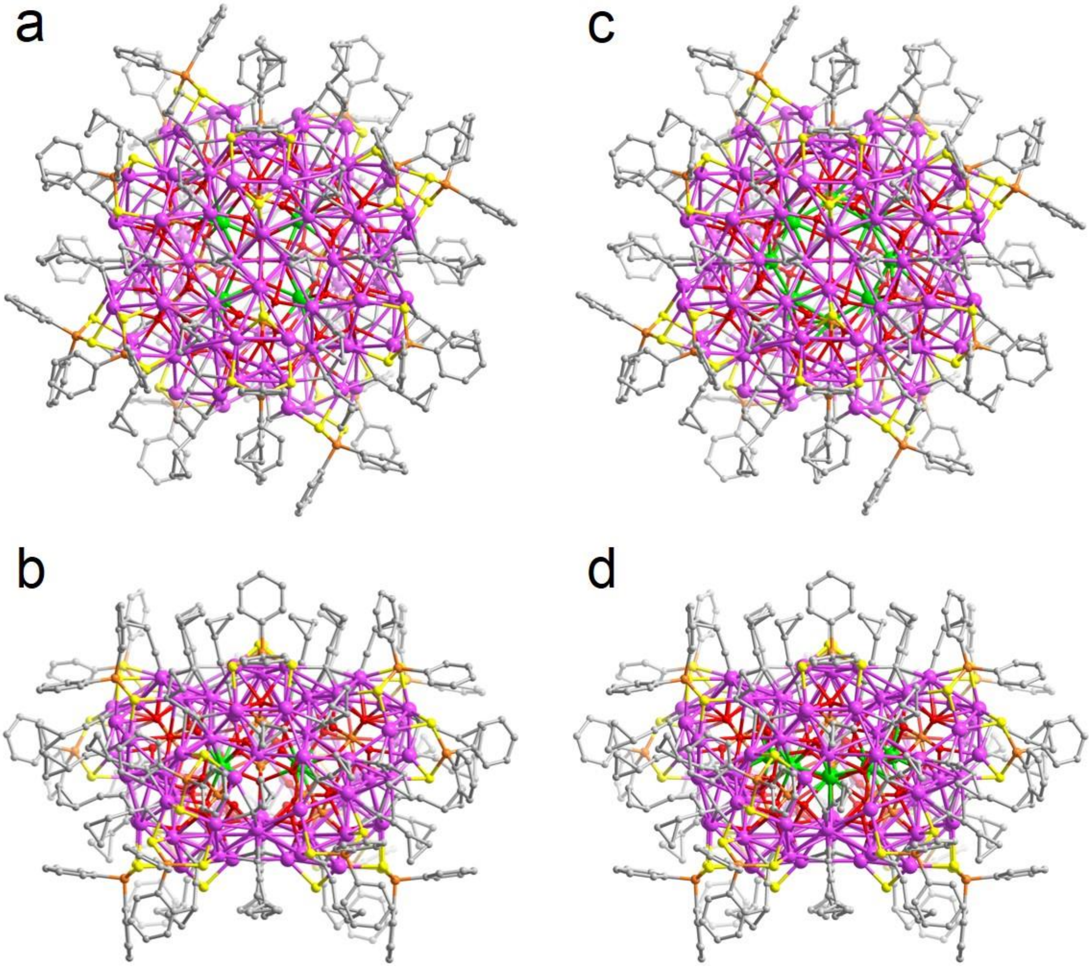

Figure S5: The top (a) and front (b) views of the total structure of Ag_{104} cluster in Ag104c·Ag108b. The top (c) and front (d) views of the total structure of Ag_{108} cluster in Ag104c·Ag108b. Hydrogen atoms are removed for clarity. Color legend: Ag, purple and green; As, royalblue; P, orange; S, yellow; O, red; C, gray.

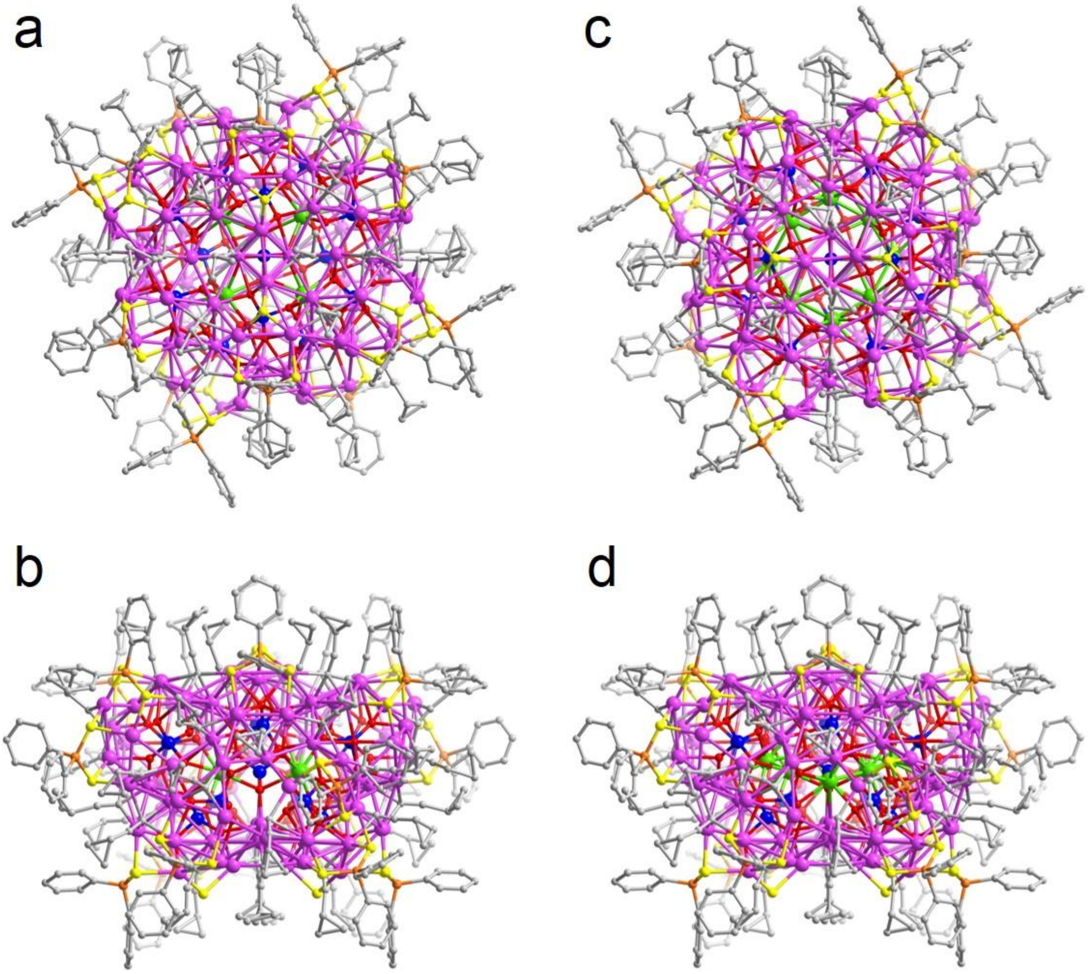

Figure S6: The IR spectrum of Ag104a.

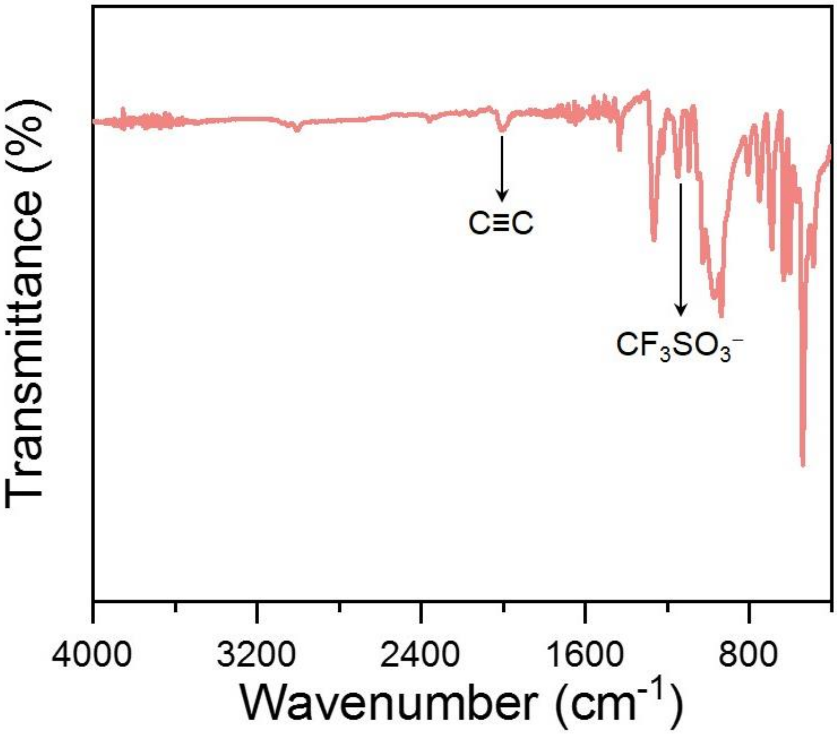

Figure S7: The UV-vis spectrum of Ag104a in CH_2Cl_2.

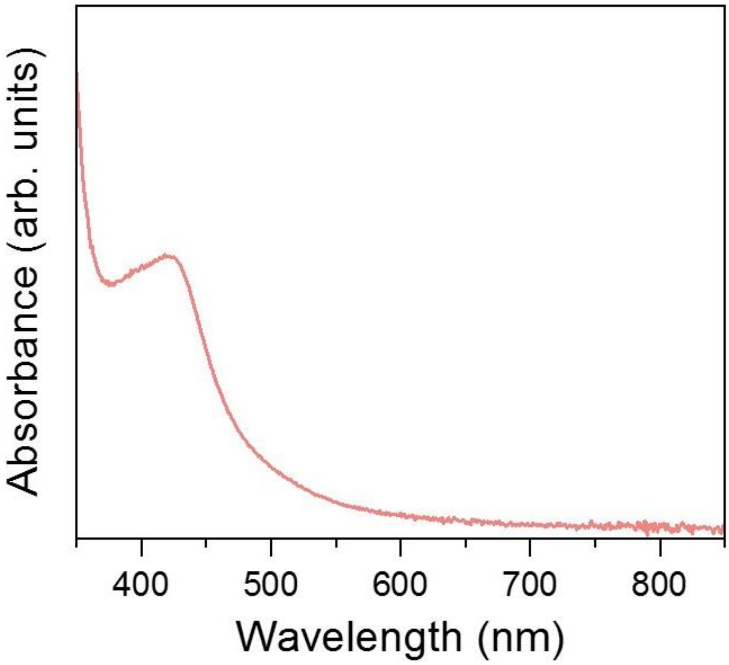

Figure S8: Total structure of Ag_{104} cluster view along two different directions. Hydrogen atoms are removed for clarity. The PO_4^{3-} anions are highlighted as blue tetrahedra. Color legend: Ag, purple; P, orange; S, yellow; O, red; C, gray.

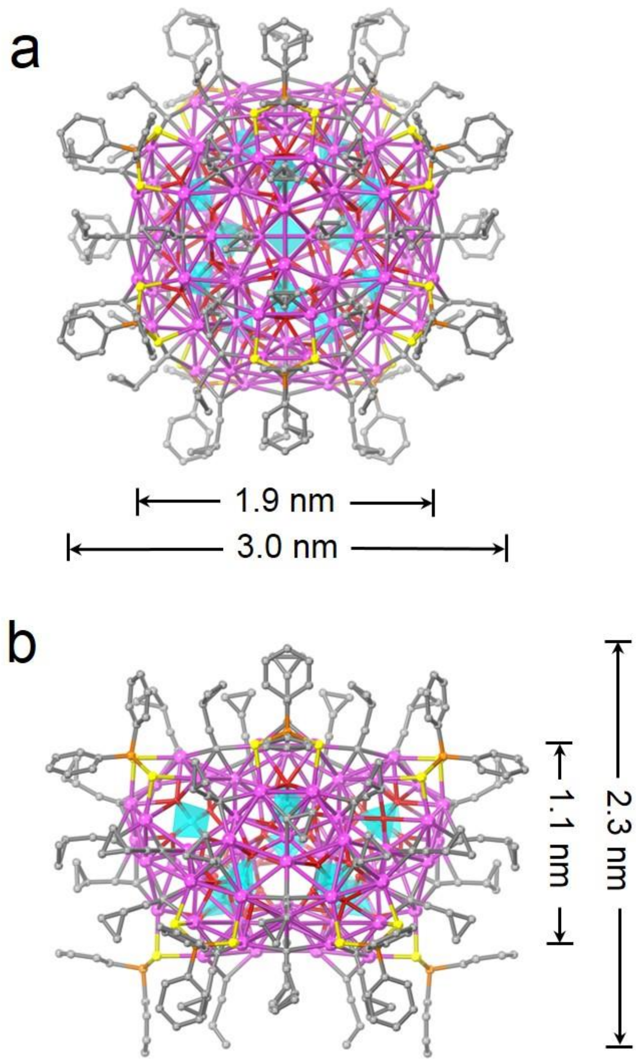

Figure S9: The negative-ion mode ESI-MS information for Ag104a dissolved in CH_2Cl_2.

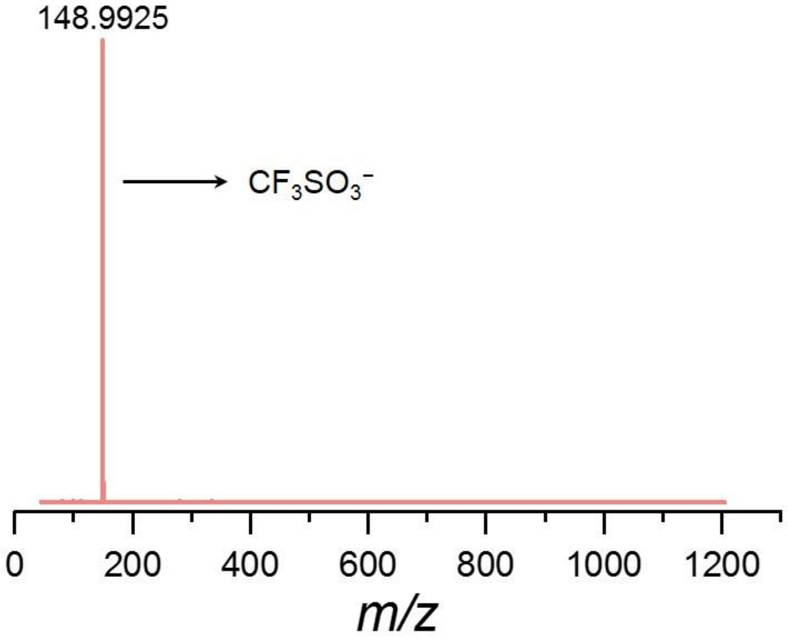

Figure S10: ^{31}P NMR analysis of Ag104a and Ag104b·Ag108a in CD_2Cl_2.

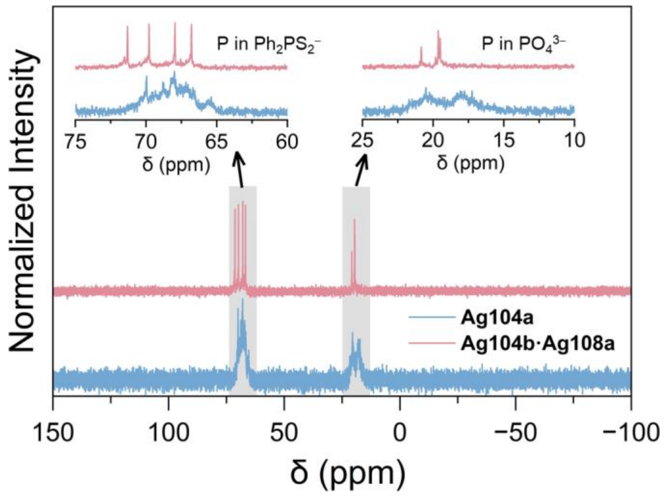

Figure S11: ^{1}H NMR analysis of Ag104a (a) and Ag104b·Ag108a (b) in CD_2Cl_2.

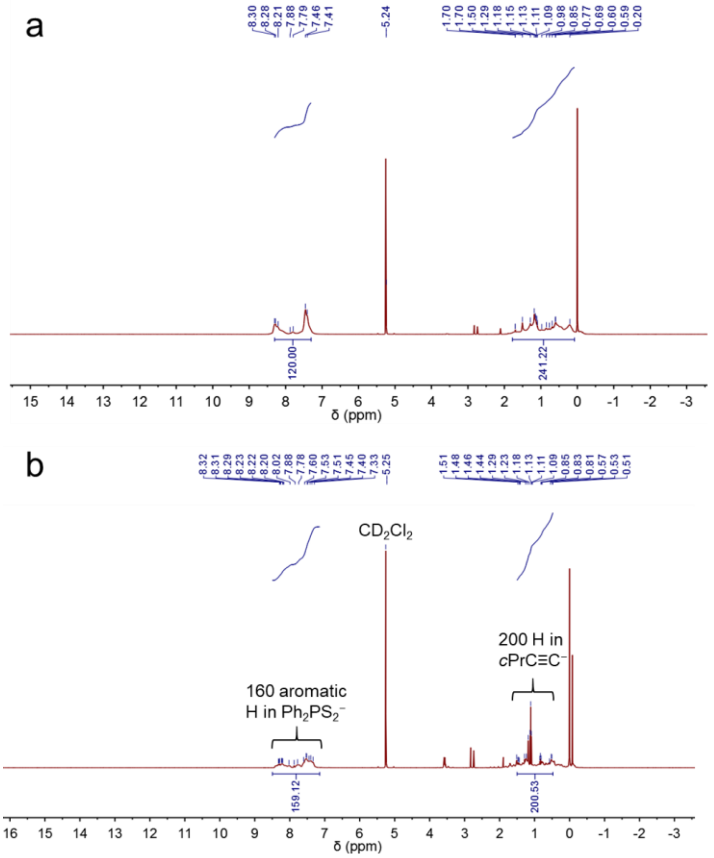

Figure S12: The two Ag_{14} units containing two Ag_4 tetrahedra in Ag104a.

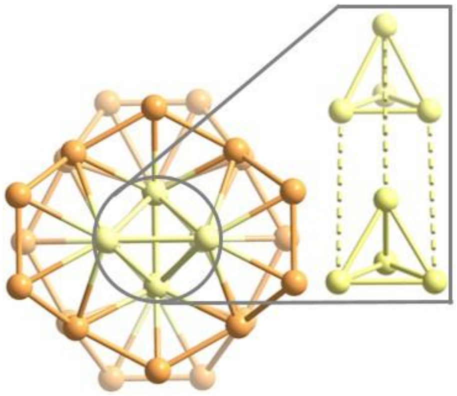

Figure S13: The entire silver skeleton of Ag_{104} cluster also can be a distorted Ag_4 square wrapped by an integrated Ag_{100} shell. Dashed lines indicate that the Ag···Ag distances longer than 3.44 Å corresponding to the absence of argentophilic interaction.

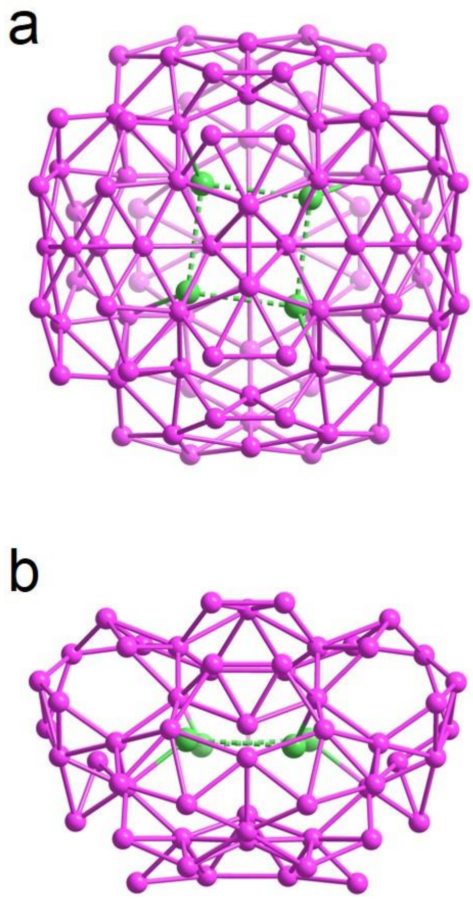

Figure S14: The coordination mode of the innermost PO_4^{3-} anion in Ag104a. Color legend: Ag, green; P, orange; O, red.

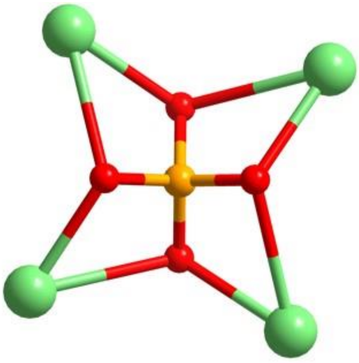

μ_8-κ^2:κ^2:κ^2:κ^2

Figure S15: Top (a) and front (b) views of the distribution of 20 *c*PrC≡C^{-} ligands at the poles on the Ag_{100} shell of Ag104a. (c) Three coordination modes in a ratio of 3:1:1 capping at the opposite two poles. Top (d) and front (e) views of the distribution of 28 *c*PrC≡C^{-} ligands near to the equator on the Ag_{100} shell of Ag104a. (f) Three coordination modes in a ratio of 3:3:1 of the *c*PrC≡C^{-} ligands distributed near to the equator. Color legend: Ag, purple; C, gray, pale blue, light green, and black.

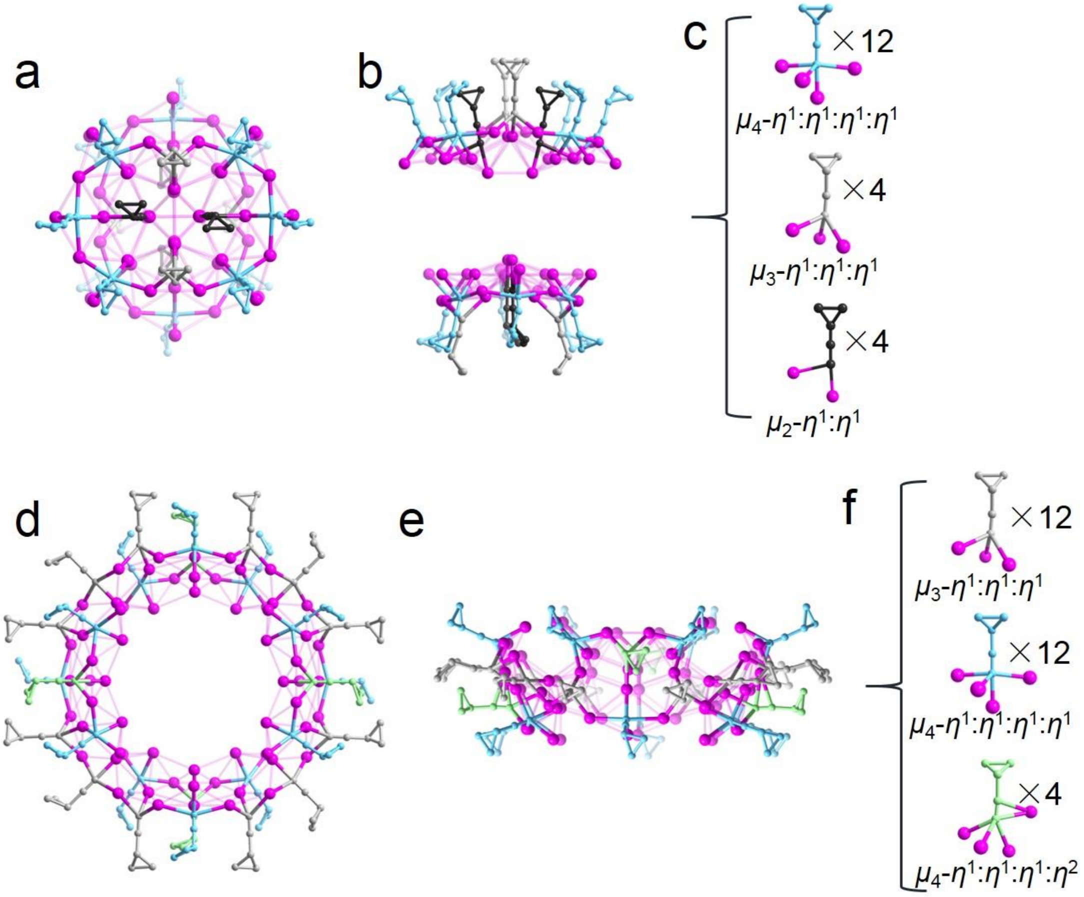

Figure S16: Top (a) and front (b) views of the distribution and coordination modes of $Ph_2PS_2^-$ on the Ag_{100} shell of Ag104a Color legend: Ag, purple; P, orange; S, yellow; C, gray.

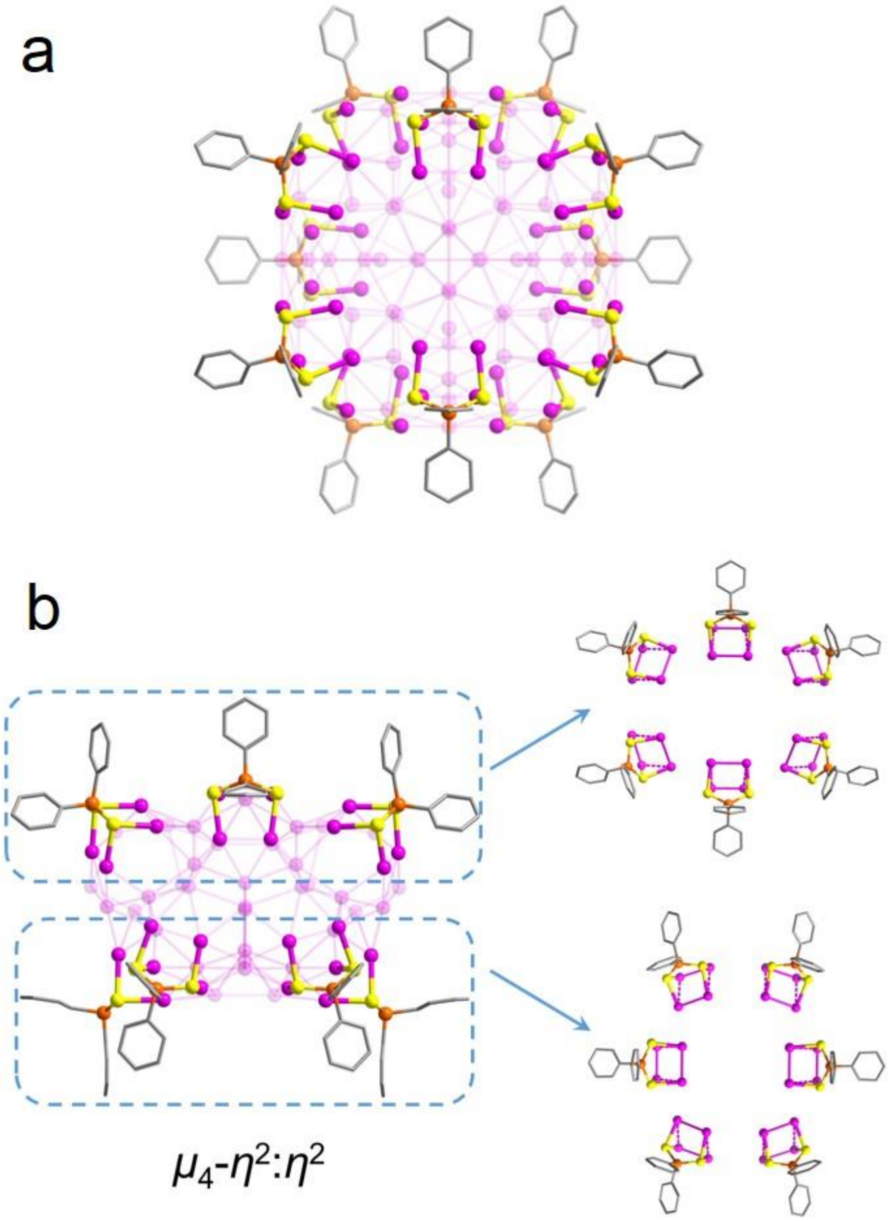

Figure S17: Top (a) and front (b) views of the silver-containing $(PO_4)@Ag_4@(PO_4)_{12}$ template layer of Ag_{104} cluster in Ag104b·Ag108a. Top (c) and front (d) views of the silver-containing $(PO_4)@Ag_8@(PO_4)_{12}$ template layer of Ag_{108} cluster in Ag104b·Ag108a. The PO_4^{3-} anions are highlighted as pale yellow tetrahedron. Color legend: Ag, green; P, orange; O, red.

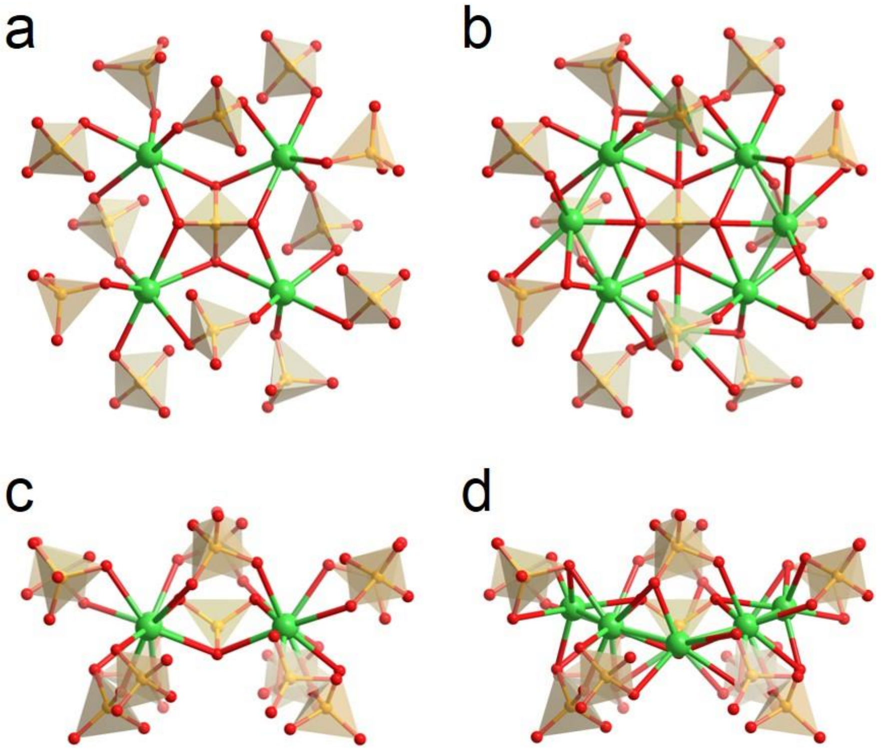

Figure S18: (a) Top view of the Ag_{76} consisting of an Ag_4 square encircled by an Ag_{72} garland in Ag_{104} cluster of Ag104b·Ag108a. (b) Top view of the Ag_{80} consisting of an Ag_8 unit encircled by an Ag_{72} garland in Ag_{108} cluster of Ag104b·Ag108a. The whole silver skeleton of Ag_{104} (c) and Ag_{108} clusters in Ag104b·Ag108a (d). (e) Two Ag_{14} units containing Ag_4 tetrahedron (yellow). Dashed lines indicate that the Ag···Ag distances longer than 3.44 Å corresponding to the absence of argentophilic interaction.

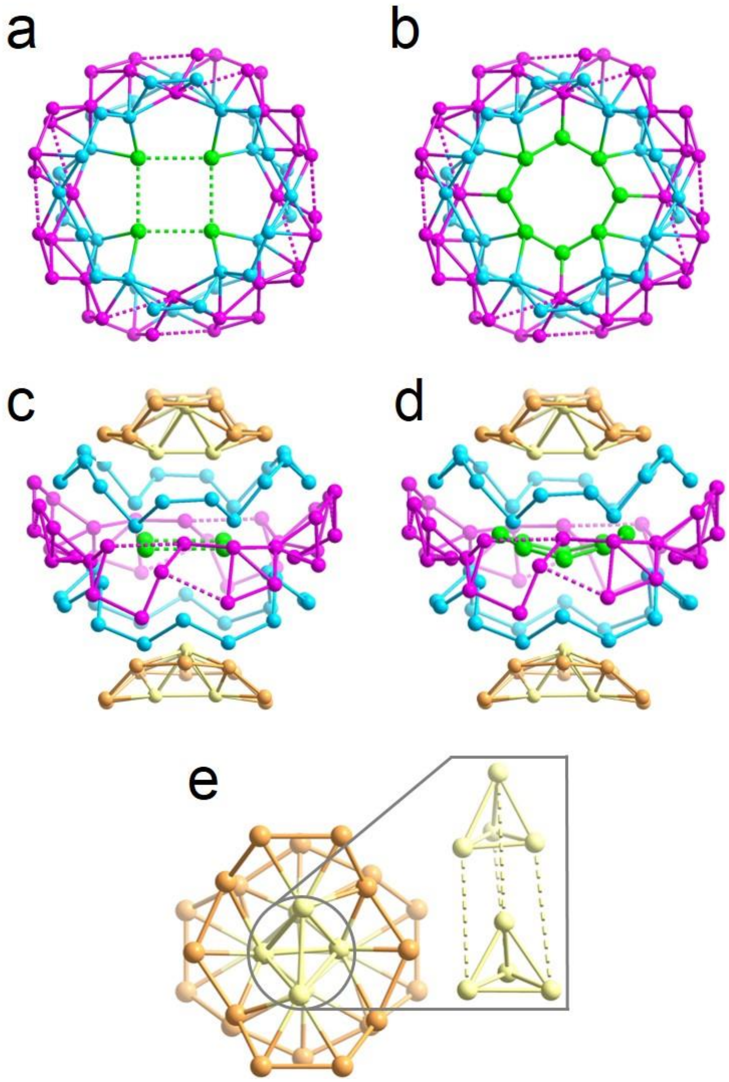

Figure S19: The coordination modes of the most central PO_4^{3-} in Ag_{104} cluster of Ag104b·Ag108a (a) and Ag_{108} cluster of Ag104b·Ag108a (b) respectively. Color legend: Ag, green; P, orange; O, red.

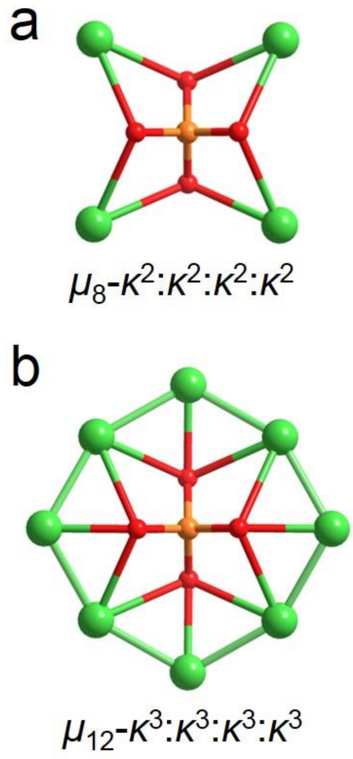

Figure S20: Top (a) and front (b) views of the distribution of 16 *c*PrC≡C^{-} and 4 S^{2-} ligands at the poles on the Ag_{100} shell of both Ag_{104} and Ag_{108} clusters in Ag104b·Ag108a. (c) The coordination patterns and number of *c*PrC≡C^{-} and S^{2-} ligands located at the poles. Top (d) and front (e) views of the distribution of 24 *c*PrC≡C^{-} ligands near to the equator on the Ag_{100} shell of both Ag_{104} and Ag_{108} clusters in Ag104b·Ag108a. (f) Two coordination modes of the *c*PrC≡C^{-} ligands distributed near to the equator. Color legend: Ag, purple; C, gray, pale blue and light green.

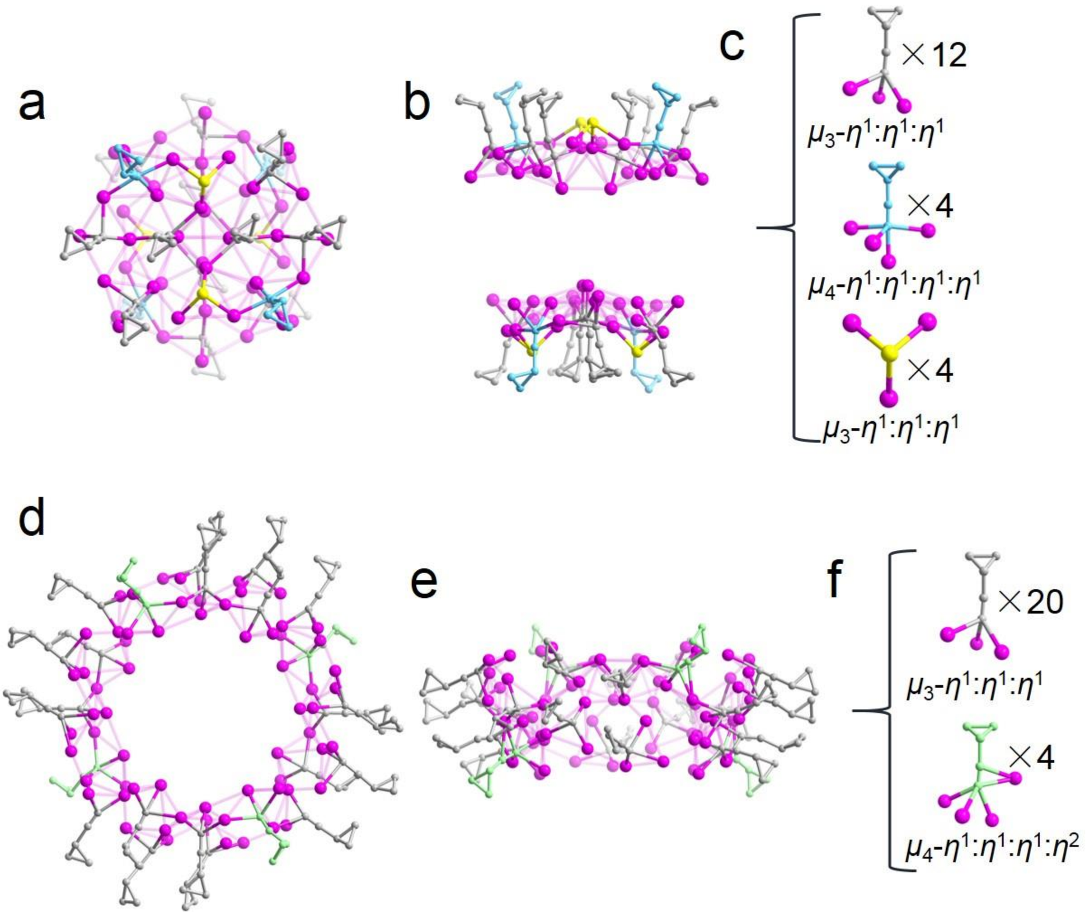

Figure S21: Top (a) and front (b) views of the distribution and the coordination mode of $Ph_2PS_2^-$ on the Ag_{100} shell of both Ag_{104} and Ag_{108} clusters in Ag104b·Ag108a. Color legend: Ag, purple; P, orange; S, yellow; C, gray.

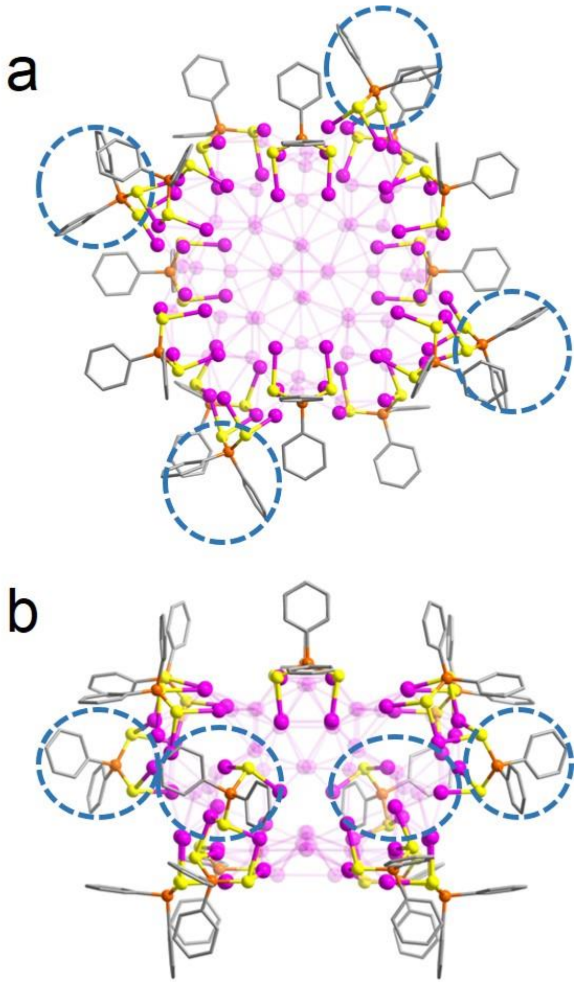

Figure S22: The +5 species of Ag104a in CH_2Cl_2 identified by ESI-MS. (a) The zoom-in experimental (blue lines) and simulated (pink lines) mass spectra for each labeled species. (b) The assigned formulas for 5a–5n.

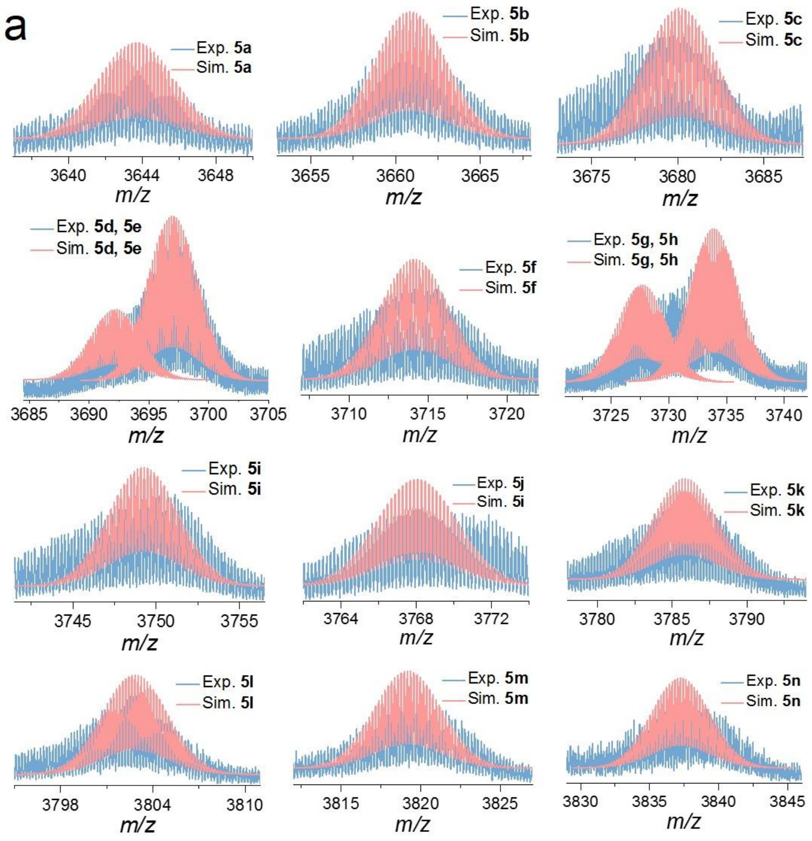

b

Peak	Species	Exp. m/z	Sim. m/z
**5a**	$[Ag_{104}(PO_4)_{13}(c\text{PrC}{\equiv}C)_{50}(Ph_2PS_2)_{10}(H_2O)]^{5+}$	3643.75	3643.69
**5b**	$[Ag_{104}(PO_4)_{13}(c\text{PrC}{\equiv}C)_{50}(Ph_2PS_2)_{10}(H_2O)(CH_2Cl_2)]^{5+}$	3660.73	3660.68
**5c**	$[Ag_{104}(PO_4)_{13}(c\text{PrC}{\equiv}C)_{49}(Ph_2PS_2)_{11}(H_2O)]^{5+}$	3680.54	3680.48
**5d**	$[Ag_{104}(PO_4)_{13}(c\text{PrC}{\equiv}C)_{48}(Ph_2PS_2)_{11}Cl(CH_2Cl_2)(H_2O)]^{5+}$	3691.75	3691.66
**5e**	$[Ag_{104}(PO_4)_{13}(c\text{PrC}{\equiv}C)_{49}(Ph_2PS_2)_{11}(CH_2Cl_2)(H_2O)]^{5+}$	3697.55	3697.47
**5f**	$[Ag_{104}(PO_4)_{13}(c\text{PrC}{\equiv}C)_{48}(Ph_2PS_2)_{12}]^{5+}$	3713.92	3713.87
**5g**	$[Ag_{104}(PO_4)_{13}(c\text{PrC}{\equiv}C)_{48}(Ph_2PS_2)_{12}(H_2O)_3]^{5+}$	3727.92	3727.88
**5h**	$[Ag_{104}(PO_4)_{13}(c\text{PrC}{\equiv}C)_{48}(Ph_2PS_2)_{12}(CH_2Cl_2)(H_2O)]^{5+}$	3734.53	3734.46
**5i**	$[NaAg_{104}(PO_4)_{13}(c\text{PrC}{\equiv}C)_{48}(Ph_2PS_2)_{12}(CF_3SO_3)]^{5+}$	3748.31	3748.26
**5j**	$[NaAg_{104}(PO_4)_{13}(c\text{PrC}{\equiv}C)_{48}(Ph_2PS_2)_{12}(CF_3SO_3)(CH_2Cl_2)(H_2O)]^{5+}$	3768.90	3768.85
**5k**	$[Na_2Ag_{104}(PO_4)_{13}(c\text{PrC}{\equiv}C)_{48}(Ph_2PS_2)_{12}(CF_3SO_3)_2(H_2O)]^{5+}$	3786.30	3786.25
**5l**	$[Na_2Ag_{104}(PO_4)_{13}(c\text{PrC}{\equiv}C)_{48}(Ph_2PS_2)_{12}(CF_3SO_3)_2(H_2O)(CH_2Cl_2)]^{5+}$	3803.29	3803.24
**5m**	$[Na_2Ag_{104}(PO_4)_{13}(c\text{PrC}{\equiv}C)_{48}(Ph_2PS_2)_{12}(CF_3SO_3)_2(H_2O)(CH_2Cl_2)_2]^{5+}$	3820.29	3820.23
**5n**	$[Na_2Ag_{104}(PO_4)_{13}(c\text{PrC}{\equiv}C)_{48}(Ph_2PS_2)_{12}(CF_3SO_3)_2(H_2O)(CH_2Cl_2)_3]^{5+}$	3837.27	3837.22

Figure S23: (a) The zoom-in mass spectrum of experimental (blue lines) and simulated (pink lines) isotope patterns for +4 labeled species of Ag104a (b) The assigned formulas for 4a–4m.

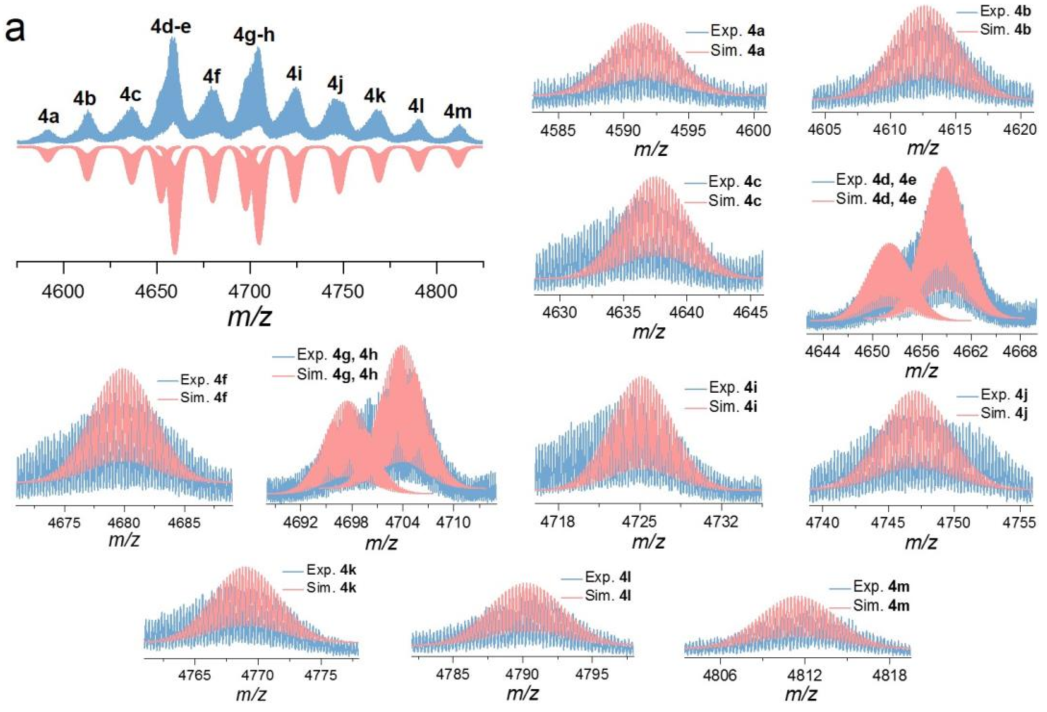

b

Peak	Species	Exp. m/z	Sim. m/z
4a	$[Ag_{104}(PO_4)_{13}(c\text{PrC≡C})_{47}(Ph_2PS_2)_{12}(CF_3SO_3)(H_2O)]^{4+}$	4591.90	4591.85
4b	$[Ag_{104}(PO_4)_{13}(c\text{PrC≡C})_{50}(Ph_2PS_2)_{10}(CF_3SO_3)(H_2O)(CH_2Cl_2)]^{4+}$	4613.14	4613.09
4c	$[Ag_{104}(PO_4)_{13}(c\text{PrC≡C})_{49}(Ph_2PS_2)_{11}(CF_3SO_3)(H_2O)]^{4+}$	4638.15	4638.09
4d	$[Ag_{104}(PO_4)_{13}(c\text{PrC≡C})_{48}(Ph_2PS_2)_{12}Cl]^{4+}$	4651.14	4651.08
4e	$[Ag_{104}(PO_4)_{13}(c\text{PrC≡C})_{49}(Ph_2PS_2)_{12}]^{4+}$	4658.66	4658.60
4f	$[Ag_{104}(PO_4)_{13}(c\text{PrC≡C})_{48}(Ph_2PS_2)_{12}(CF_3SO_3)]^{4+}$	4679.63	4679.58
4g	$[Ag_{104}(PO_4)_{13}(c\text{PrC≡C})_{48}(Ph_2PS_2)_{12}(CF_3SO_3)(H_2O)_3]^{4+}$	4697.12	4697.08
4h	$[Ag_{104}(PO_4)_{13}(c\text{PrC≡C})_{48}(Ph_2PS_2)_{13}]^{4+}$	4704.63	4704.59
4i	$[Ag_{104}(PO_4)_{13}(c\text{PrC≡C})_{48}(Ph_2PS_2)_{13}(CH_2Cl_2)]^{4+}$	4725.86	4725.82
4j	$[Ag_{104}(PO_4)_{13}(c\text{PrC≡C})_{48}(Ph_2PS_2)_{13}(CH_2Cl_2)_2]^{4+}$	4747.12	4747.06
4k	$[NaAg_{104}(PO_4)_{13}(c\text{PrC≡C})_{48}(Ph_2PS_2)_{13}(CF_3SO_3)(CH_2Cl_2)]^{4+}$	4768.86	4768.81
4l	$[NaAg_{104}(PO_4)_{13}(c\text{PrC≡C})_{48}(Ph_2PS_2)_{13}(CF_3SO_3)(CH_2Cl_2)_2]^{4+}$	4790.10	4790.05
4m	$[NaAg_{104}(PO_4)_{13}(c\text{PrC≡C})_{48}(Ph_2PS_2)_{13}(CF_3SO_3)(CH_2Cl_2)_3]^{4+}$	4811.33	4811.29

Figure S24: (a) The zoom-in mass spectrum of experimental (blue lines) and simulated (pink lines) isotope patterns for +6 labeled species of Ag104a (b) The assigned formulas for 6a–6l.

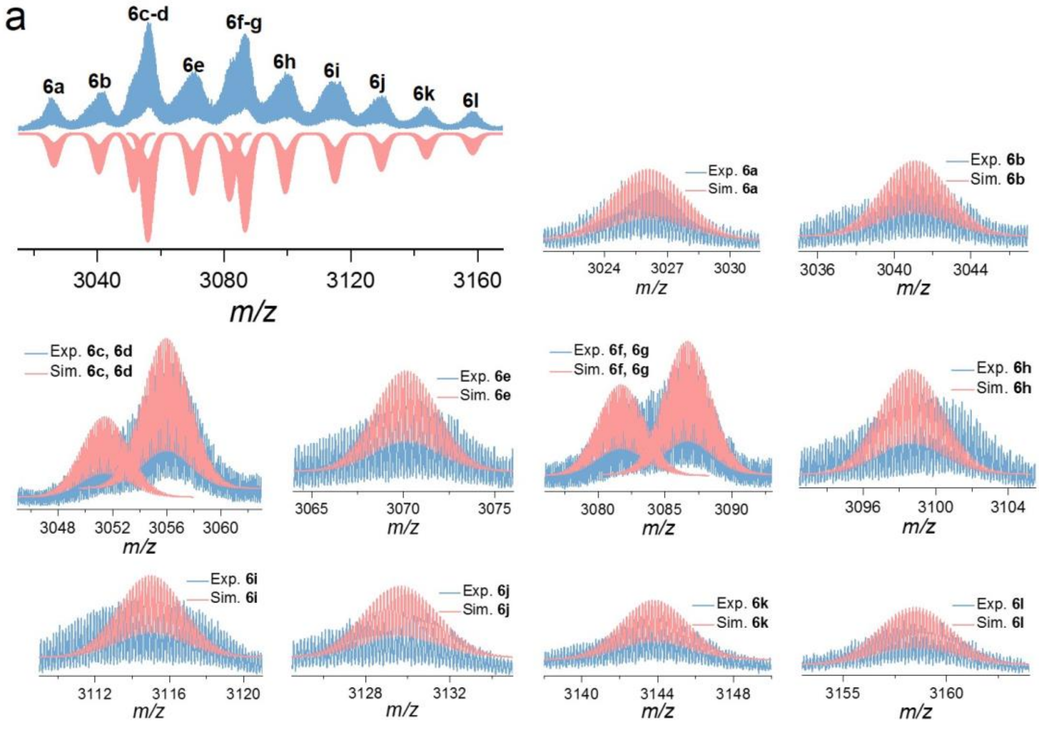

b

Peak	Species	Exp. m/z	Sim. m/z
6a	$[Ag_{104}(PO_4)_{13}(c\text{PrC}{\equiv}C)_{49}(Ph_2PS_2)_{10}(H_2O)]^{6+}$	3025.62	3025.57
6b	$[NaAg_{104}(PO_4)_{13}(c\text{PrC}{\equiv}C)_{49}(Ph_2PS_2)_{10}Cl(H_2O)_3]^{6+}$	3041.45	3041.40
6c	$[Ag_{104}(PO_4)_{13}(c\text{PrC}{\equiv}C)_{48}(Ph_2PS_2)_{10}(CF_3SO_3)(CH_2Cl_2)]^{6+}$	3050.77	3050.71
6d	$[Ag_{104}(PO_4)_{13}(c\text{PrC}{\equiv}C)_{48}(Ph_2PS_2)_{11}(H_2O)]^{6+}$	3056.29	3056.23
6e	$[NaAg_{104}(PO_4)_{13}(c\text{PrC}{\equiv}C)_{49}(Ph_2PS_2)_{11}(H_2O)]^{6+}$	3070.95	3070.90
6f	$[NaAg_{104}(PO_4)_{13}(c\text{PrC}{\equiv}C)_{48}(Ph_2PS_2)_{11}(CF_3SO_3)]^{6+}$	3080.93	3080.88
6g	$[Ag_{104}(PO_4)_{13}(c\text{PrC}{\equiv}C)_{47}(Ph_2PS_2)_{12}(H_2O)]^{6+}$	3087.11	3087.05
6h	$[NaAg_{104}(PO_4)_{13}(c\text{PrC}{\equiv}C)_{48}(Ph_2PS_2)_{12}]^{6+}$	3098.77	3098.72
6i	$[NaAg_{104}(PO_4)_{13}(c\text{PrC}{\equiv}C)_{48}(Ph_2PS_2)_{12}(CH_2Cl_2)(H_2O)]^{6+}$	3115.93	3115.88
6j	$[NaAg_{104}(PO_4)_{13}(c\text{PrC}{\equiv}C)_{48}(Ph_2PS_2)_{12}(CH_2Cl_2)_2(H_2O)]^{6+}$	3130.08	3130.04
6k	$[NaAg_{104}(PO_4)_{13}(c\text{PrC}{\equiv}C)_{48}(Ph_2PS_2)_{12}(CH_2Cl_2)_3(H_2O)]^{6+}$	3144.25	3144.20
6l	$[NaAg_{104}(PO_4)_{13}(c\text{PrC}{\equiv}C)_{48}(Ph_2PS_2)_{12}(CH_2Cl_2)_4(H_2O)]^{6+}$	3158.40	3158.36

Figure S25. The UV-Vis spectra of Ag104a in DMF solution (3.5mg/1 mL) at 24 ℃ and of 42% RH under dark condition.

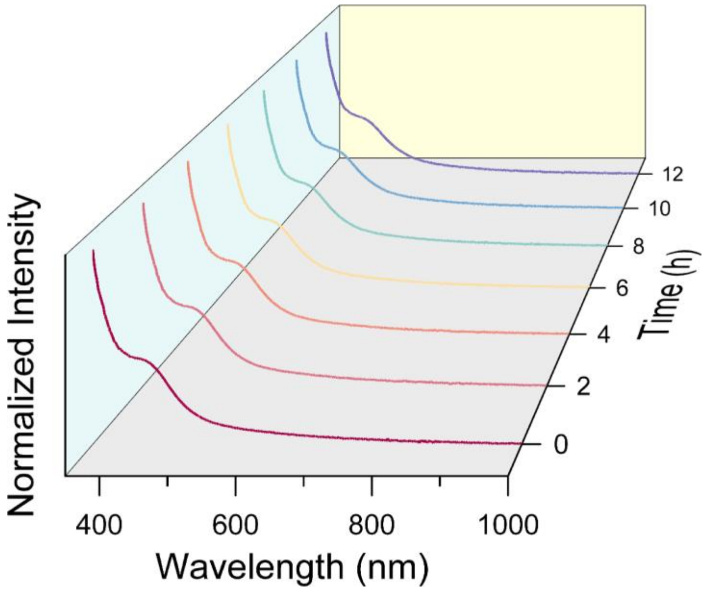

Figure S26: SAXS of Ag104a in DMF using a monodispersed sphere model. Inset: the pair-distance distribution function p(r) curve.

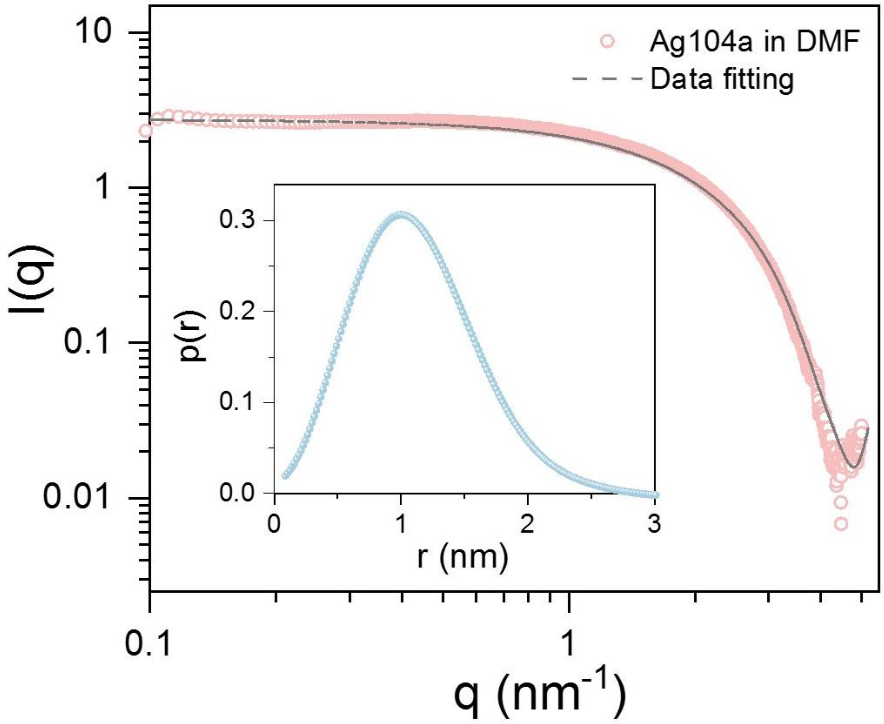

Figure S27: The +5 species of Ag104b·Ag108a in CH_2Cl_2 identified by ESI-MS. (a) The zoom-in experimental (pink lines) and simulated (blue lines) mass spectra for each labeled species. (b) The assigned formulas for 5a'–5g'.

a

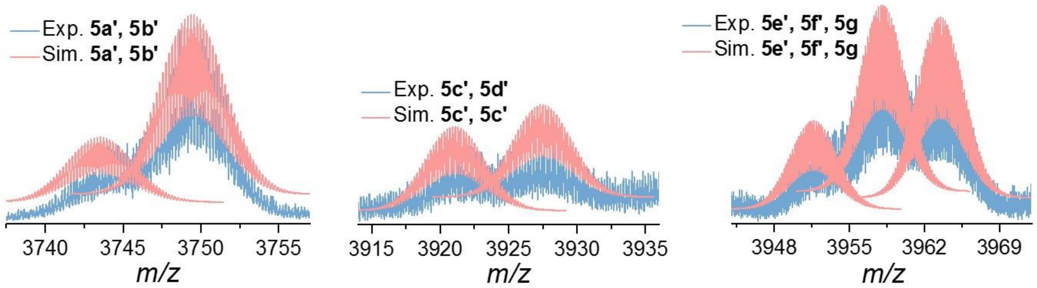

b

Peak	Species	Exp. m/z	Sim. m/z
**5a'**	$[Ag_{104}(PO_4)_{13}S_4(c\text{PrC}{\equiv}C)_{36}(Ph_2PS_2)_{15}Cl(H_2O)]^{5+}$	3743.46	3743.55
**5b'**	$[Ag_{104}(PO_4)_{13}S_4(c\text{PrC}{\equiv}C)_{37}(Ph_2PS_2)_{15}(H_2O)]^{5+}$	3749.27	3749.36
**5c'**	$[Ag_{108}(PO_4)_{13}S_4(c\text{PrC}{\equiv}C)_{40}(Ph_2PS_2)_{16}]^{5+}$	3921.02	3921.10
**5d'**	$[Ag_{108}(PO_4)_{13}S_4(c\text{PrC}{\equiv}C)_{40}(Ph_2PS_2)_{16}(CH_3OH)]^{5+}$	3927.42	3927.51
**5e'**	$[Ag_{108}(PO_4)_{13}S_4(c\text{PrC}{\equiv}C)_{40}(Ph_2PS_2)_{16}(CH_2Cl_2)(CH_3OH)(H_2O)_2]^{5+}$	3951.77	3951.71
**5f'**	$[Ag_{108}(PO_4)_{13}S_4(c\text{PrC}{\equiv}C)_{40}(Ph_2PS_2)_{16}(CH_2Cl_2)(CH_3OH)_2(H_2O)]^{5+}$	3958.18	3958.11
**5g'**	$[Ag_{108}(PO_4)_{13}S_4(c\text{PrC}{\equiv}C)_{40}(Ph_2PS_2)_{16}(CH_2Cl_2)(CH_3OH)_4]^{5+}$	3963.81	3963.72

Figure S28: (a) The zoom-in mass spectra of experimental (pink lines) and simulated (blue lines) isotope patterns for +4 labeled species of Ag104b·Ag108a. (b) The assigned formulas for 4a’–4f’.

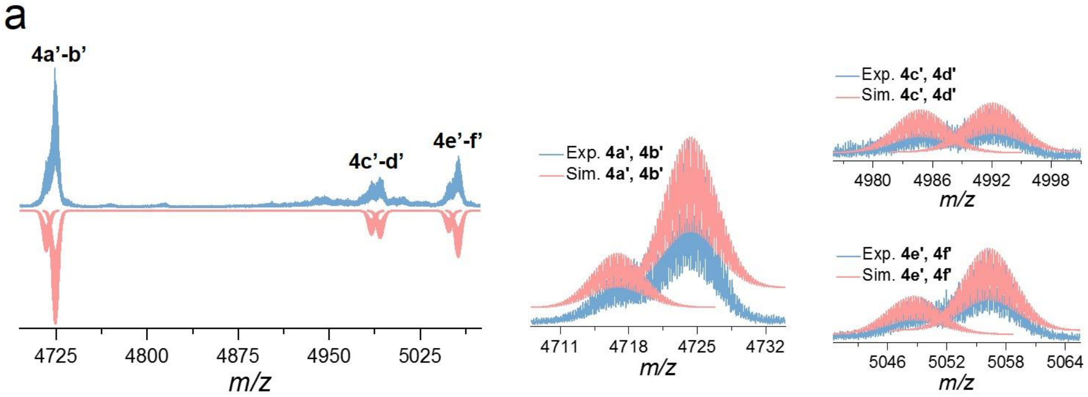

b

Peak	Species	Exp. m/z	Sim. m/z
**4a’**	$[Ag_{104}(PO_4)_{13}S_4(c\text{PrC≡C})_{37}(Ph_2PS_2)_{15}Cl(CH_2Cl_2)(H_2O)]^{4+}$	4716.84	4716.93
**4b’**	$[Ag_{104}(PO_4)_{13}S_4(c\text{PrC≡C})_{38}(Ph_2PS_2)_{15}(CH_2Cl_2)(H_2O)]^{4+}$	4724.09	4724.20
**4c’**	$[Ag_{108}(PO_4)_{13}S_4(c\text{PrC≡C})_{40}(Ph_2PS_2)_{16}(CF_3SO_3)(CH_2Cl_2)(CH_3OH)_2(H_2O)_2]^{4+}$	4984.97	4984.88
**4d’**	$[Ag_{108}(PO_4)_{13}S_4(c\text{PrC≡C})_{40}(Ph_2PS_2)_{16}(CF_3SO_3)(CH_2Cl_2)(CH_3OH)_3(H_2O)_2]^{4+}$	4992.97	4992.88
**4e’**	$[Na_2Ag_{108}(PO_4)_{13}S_4(c\text{PrC≡C})_{40}(Ph_2PS_2)_{16}(CF_3SO_3)_3(CH_3OH)_3]^{4+}$	5048.67	5048.61
**4f’**	$[Na_2Ag_{108}(PO_4)_{13}S_4(c\text{PrC≡C})_{40}(Ph_2PS_2)_{16}(CF_3SO_3)_3(CH_3OH)_4]^{4+}$	5056.71	5056.62

Figure S29: (a) The zoom-in mass spectra of experimental (pink lines) and simulated (blue lines) isotope patterns for +6 labeled species of Ag_{104} and Ag_{108} clusters in Ag104b·Ag108a. (b) The assigned formulas for 6a'–6f'.

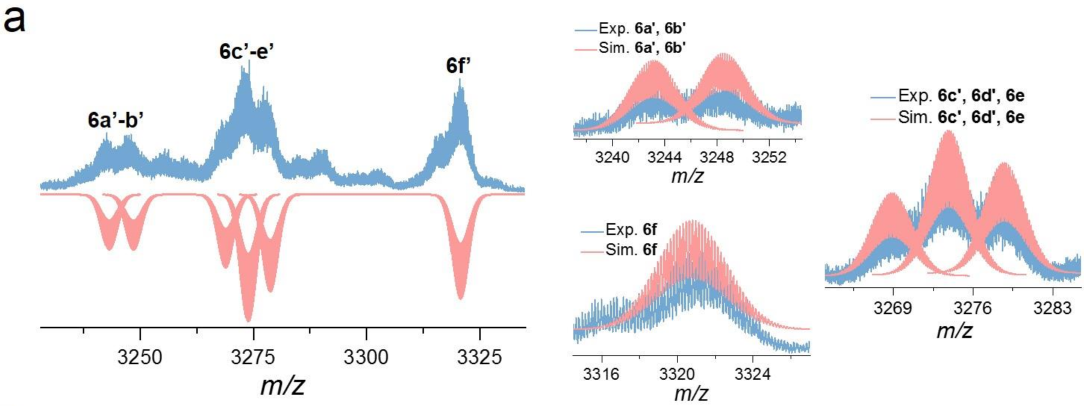

b

Peak	Species	Exp. m/z	Sim. m/z
6a'	$[Ag_{108}(PO_4)_{13}S_4(c\text{PrC≡C})_{40}(Ph_2PS_2)_{15}(CH_2Cl_2)(H_2O)]^{6+}$	3243.15	3243.08
6b'	$[Ag_{108}(PO_4)_{13}S_4(c\text{PrC≡C})_{40}(Ph_2PS_2)_{15}(CH_2Cl_2)(CH_3OH)(H_2O)]^{6+}$	3248.50	3248.42
6c'	$[Ag_{108}(PO_4)_{13}S_4(c\text{PrC≡C})_{40}(Ph_2PS_2)_{16}H]^{6+}$	3267.83	3267.76
6d'	$[Ag_{108}(PO_4)_{13}S_4(c\text{PrC≡C})_{40}(Ph_2PS_2)_{16}(H_2O)_2H]^{6+}$	3273.82	3273.76
6e'	$[Ag_{108}(PO_4)_{13}S_4(c\text{PrC≡C})_{40}(Ph_2PS_2)_{16}(CH_3OH)_2H]^{6+}$	3278.50	3278.43
6f'	$[NaAg_{108}(PO_4)_{13}S_4(c\text{PrC≡C})_{40}(Ph_2PS_2)_{16}(CH_2Cl_2)_2(CH_3OH)_2(H_2O)_2]^{6+}$	3316.48	3316.42
6g'	$[Na_2Ag_{108}(PO_4)_{13}S_4(c\text{PrC≡C})_{40}(Ph_2PS_2)_{16}(CF_3SO_3)(H_2O)_7]^{6+}$	3321.15	3321.09

Figure S30: Electronic structure for Ag104a, and the related hypothetical species obtained by removal of the central PO_4^{3-} anion. The Fermi level was set as zero for comparison between the different species, which is related to the HOMO level at 0 K.

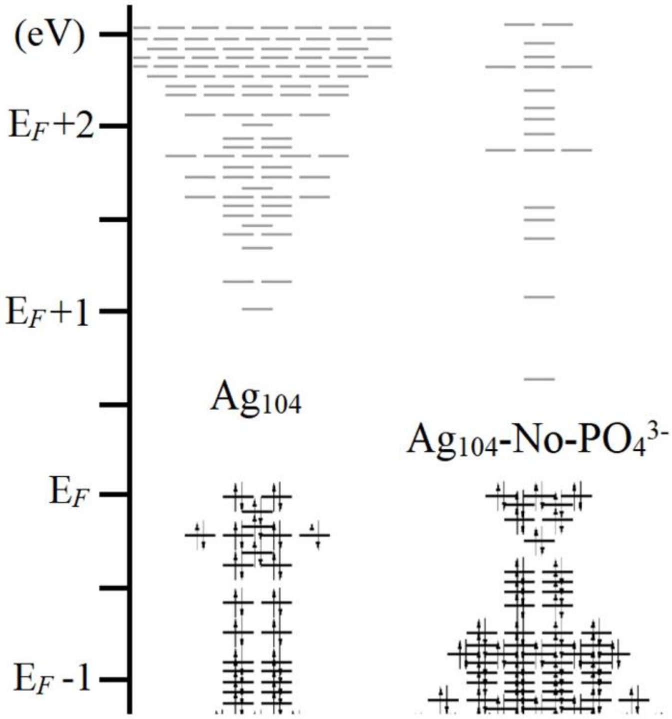

Figure S31: Calculated UV-vis absorbance profile for Ag104a, and the related hypothetical species obtained by removal of the central PO_4^{3-} anion.

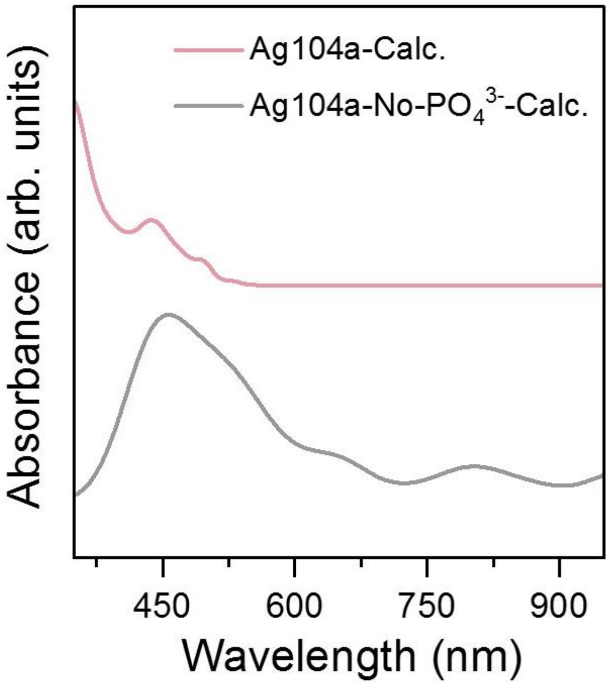

Figure S32: (a) UV-vis spectrum of Ag104a in the solid state. (b) UV-vis spectrum of Kubelka-Munk function *vs* energy (eV) and Tauc plots of Ag104a.

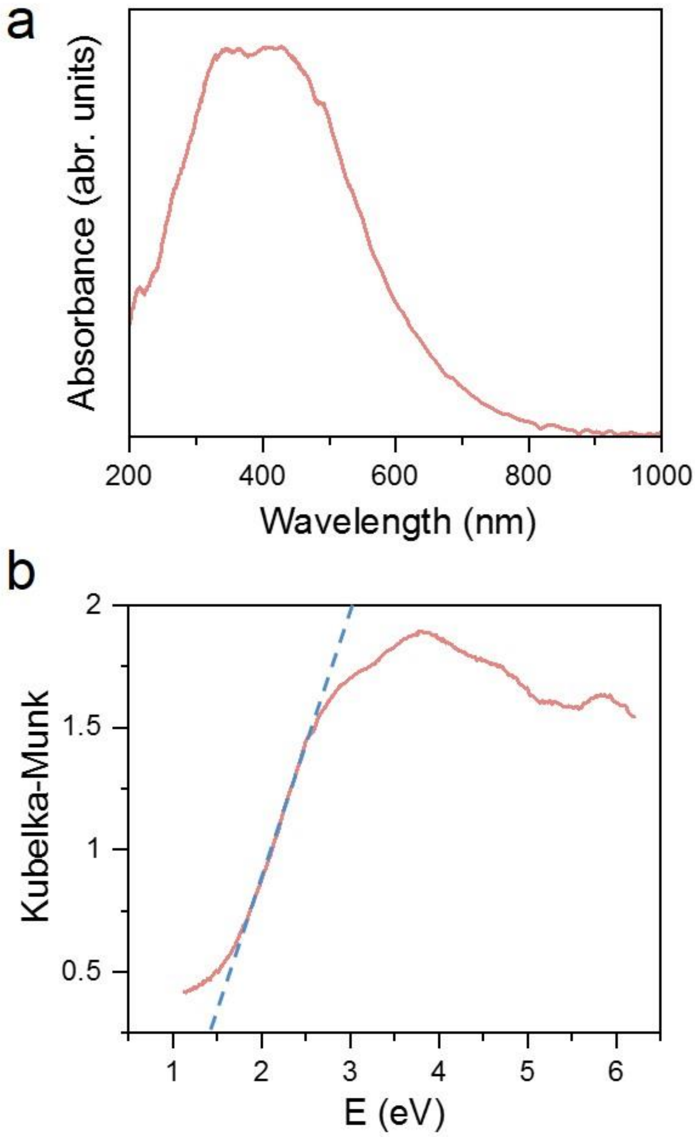

Figure S33: The electron paramagnetic resonance (EPR) spectra of Ag104a in the presence of TEMP.

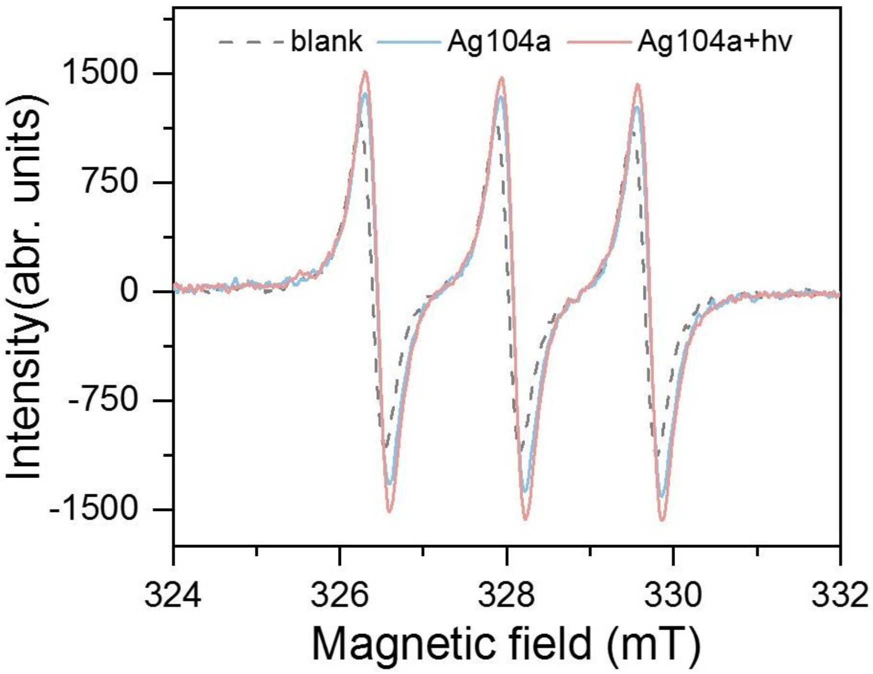

Table S1: Summary of the solution pH values before and after the solvothermal reaction in the synthesis of Ag104a and Ag104b ·Ag108a.

Template	Alkali	pH value before reaction	pH value after reaction	Product
Na_3PO_4	None	7.95	7.91	**Ag104a**
	TMEDA (20 μL)	8.55	8.47	**Ag104a**
	Et_3N (20 μL)	9.38	9.02	**Ag104a**
	$NH_3{\cdot}H_2O$ (20 μL)	10.65	10.40	**Ag104a**
NaH_2PO_4	None	6.65	6.72	**No crystal**
	TMEDA (20 μL)	7.74	7.63	**Ag104b ·Ag108a**
	Et_3N (20 μL)	8.62	8.54	**Ag104a**
	$NH_3{\cdot}H_2O$ (20 μL)	10.17	9.87	**Ag104a**
	Et_3N (7.5 μL)	7.68	7.55	**Ag104b ·Ag108a**
	$NH_3{\cdot}H_2O$ (2.5 μL)	7.72	7.64	**Ag104b ·Ag108a**

Table S2: Crystal data and structure refinements for Ag104a.

Identification code	**Ag104a**
Empirical formula	$C_{384}H_{360}Ag_{104}O_{52}P_{25}S_{24}$
Formula weight	18568.87
Temperature/K	173
Crystal system	tetragonal
Space group	$P4_2$/nmc
a/Å	33.0371 (5)
b/Å	33.0371 (5)
c/Å	26.0764 (14)
α/ °	90
β/ °	90
γ/ °	90
Volume/Å^3	28461.1 (18)
Z	2
ρ_{calc}g/cm^3	2.167
μ/mm^{-1}	3.685
F(000)	17454.0
Radiation	Mo Kα (λ = 0.71073)
2Θ range for data collection/ °	4.712 to 52.74
Index ranges	$-29 \leq h \leq 38$, $-39 \leq k \leq 40$, $-30 \leq l \leq 31$
Reflections collected	87980
Independent reflections	14365 [R_{int} = 0.0886, R_{sigma} = 0.0623]
Data/restraints/parameters	14365/817/896
Goodness-of-fit on F^2	1.046
Final R indexes [I>=2σ (I)]	R_1 = 0.1169, wR_2 = 0.2861
Final R indexes [all data]	R_1 = 0.1699, wR_2 = 0.3238
Largest diff. peak/hole / e Å^{-3}	4.04/-3.12

Table S3: Crystal data and structure refinements for Ag104b·Ag108a.

Identification code	**Ag104b·Ag108a**
Empirical formula	$C_{392}H_{360}Ag_{106}O_{52}P_{29}S_{36}$
Formula weight	19402.85
Temperature/K	153
Crystal system	tetragonal
Space group	*I*-4
a/Å	28.7983 (17)
b/Å	28.7983 (17)
c/Å	36.706 (3)
α/ °	90
β/ °	90
γ/ °	90
Volume/Å^3	30442 (5)
Z	2
ρ_{calc}g/cm^3	2.117
μ/mm^{-1}	28.888
F(000)	18250.0
Radiation	Cu Kα (λ = 1.54178)
2Θ range for data collection/°	6.138 to 133.962
Index ranges	$-34 \le h \le 31$, $-34 \le k \le 30$, $-43 \le l \le 43$
Reflections collected	96584
Independent reflections	26565 [R_{int} = 0.0634, R_{sigma} = 0.0521]
Data/restraints/parameters	26565/887/1353
Goodness-of-fit on F^2	1.071
Final R indexes [I>=2σ (I)]	R_1 = 0.0597, wR_2 = 0.1724
Final R indexes [all data]	R_1 = 0.0723, wR_2 = 0.1825
Largest diff. peak/hole / e Å^{-3}	1.68/-0.82
Flack parameter	0.481 (4)

Table S4: Crystal data and structure refinements for Ag104c ·Ag108b.

Identification code	**Ag104c ·Ag108b**
Empirical formula	$C_{392}H_{360}Ag_{106}As_{13}O_{52}P_{16}S_{36}$
Formula weight	19960.64
Temperature/K	173
Crystal system	tetragonal
Space group	*I*-4
a/Å	28.653 (3)
b/Å	28.653 (3)
c/Å	37.053 (6)
α/ °	90
β/ °	90
γ/ °	90
Volume/Å^3	30420 (8)
Z	2
ρ_{calc}g/cm^3	2.179
μ/mm^{-1}	29.338
F(000)	18710.0
Radiation	Cu Kα (λ = 1.54178)
2Θ range for data collection/°	3.898 to 128.188
Index ranges	$-33 \leq h \leq 33$, $-32 \leq k \leq 32$, $-43 \leq l \leq 41$
Reflections collected	91809
Independent reflections	23884 [R_{int} = 0.1216, R_{sigma} = 0.1209]
Data/restraints/parameters	23884/1178/1422
Goodness-of-fit on F^2	1.046
Final R indexes [I>=2σ (I)]	R_1 = 0.0921, wR_2 = 0.2134
Final R indexes [all data]	R_1 = 0.1474, wR_2 = 0.2538
Largest diff. peak/hole / e Å^{-3}	1.98/-1.88
Flack parameter	0.296 (5)

Table S5: Selected bond distances (Å) and angles (°) for Ag104a.

Ag1—Ag6	3.251 (7)	Ag11—Ag12	3.148 (3)
Ag1—O3	2.30 (3)	Ag11—Ag12	2.910 (4)
Ag1—O5	2.32 (2)	Ag11—C17^{iii}	2.21 (7)
Ag1—O8	2.17 (3)	Ag11—C36	2.23 (5)
Ag1^{iv}—O1	2.355 (12)	Ag12—Ag13	3.030 (2)
Ag1^{iv}—O3	2.53 (2)	Ag12^{ii}—Ag13	3.030 (2)
Ag1^{iv}—O7	2.51 (3)	Ag12—Ag14	3.143 (2)
Ag2^{iii}—Ag10	3.115 (5)	Ag12—S1	2.580 (6)
Ag2^{iii}—Ag11	3.312 (8)	Ag12—C36	2.450 (7)
Ag2—C17	2.09 (8)	Ag12—C41	2.39 (3)
Ag2—C22	2.35 (6)	Ag12—O6	2.45 (3)
Ag2—C23	2.38 (5)	Ag13—Ag14	3.014 (2)
Ag3—Ag6	2.997 (3)	Ag13—Ag14^{ii}	3.014 (2)
Ag3^{iv}—Ag7	3.242 (3)	Ag13—O8	2.34 (3)
Ag3^{iv}—Ag8	3.026 (4)	Ag11—C31	2.47 (4)
Ag3^{iv}—Ag10	3.220 (4)	Ag11—C36	2.18 (4)
Ag3—O9^{iv}	2.43 (3)	Ag12—Ag12^{vi}	3.252 (4)
Ag3—C46	2.34 (3)	Ag12—Ag12^{iv}	3.246 (4)
Ag3—C47	2.70 (3)	Ag12—Ag13	3.076 (3)
Ag4—Ag1	2.984 (9)	Ag12—S8	2.479 (3)
Ag4—Ag6	3.220 (2)	Ag12—S10	2.913 (5)
Ag4—O5	2.42 (3)	Ag12—S14	2.460 (6)
Ag4—O9^{vi}	2.26 (3)	Ag12—O17	2.525 (15)
Ag4—O9^{iv}	2.26 (3)	Ag13—Ag14	2.933 (3)
Ag4—C22	2.31 (5)	Ag13—Ag15	2.944 (3)
Ag5—Ag5^{v}	2.813 (3)	Ag13—Ag19	2.930 (3)
Ag5—Ag6	2.850 (2)	Ag13—S7	2.520 (5)
Ag5—S3	2.552 (6)	Ag13—S14	2.504 (6)
Ag5—C22	2.33 (4)	Ag14—Ag15	3.1104 (19)
Ag6—Ag7	3.133 (2)	Ag14—Ag16	2.902 (2)
Ag6—Ag9	2.954 (2)	Ag14—Ag17	3.371 (2)
Ag6—Ag14	3.088 (3)	Ag14—O2	2.34 (2)
Ag6—O3	2.26 (2)	Ag14—O3	2.40 (3)
Ag6—O5	2.27 (2)	Ag14—C41	2.17 (3)
Ag7—Ag8	2.864 (3)	Ag15—Ag16	3.337 (3)
Ag7—Ag8^{iv}	2.864 (3)	Ag15—Ag16^{i}	3.337 (3)
Ag7—O7	2.28 (3)	Ag15—O1	2.127 (16)
Ag7—C12	2.04 (5)	Ag15—O2	2.52 (2)
Ag8—S2	2.528 (11)	Ag15—C31	2.08 (3)
Ag8—C1^{iv}	2.31 (7)	Ag16—Ag16^{i}	3.311 (6)
Ag8—C12	2.461 (11)	Ag16—Ag17	3.199 (3)

Ag9—Ag12	2.989 (2)	Ag16—O2	2.31 (3)
Ag9—S2	2.593 (7)	Ag16—C27	2.23 (5)
Ag9—O6	2.48 (3)	Ag16—C31	2.65 (3)
Ag9—C41	2.37 (4)	Ag16—C31^{i}	2.65 (3)
Ag9—C46	2.43 (3)	Ag17—Ag17^{v}	2.970 (3)
Ag10—Ag11	2.984 (3)	Ag17—S3	2.573 (6)
Ag10^{ii}—Ag11	2.983 (3)	Ag17—O4	2.585 (16)
Ag10—S1	2.568 (9)	Ag17—C27	2.29 (4)
Ag10—C1^{iv}	2.30 (7)	Ag17—C21	2.46 (3)
Ag10—C17^{iii}	2.535 (14)	C41—Ag9—C46	120.4 (10)
O1^{vi}—Ag1—O7^{iv}	143.8 (9)	C41—Ag9—O6	95.4 (10)
O1^{vi}—Ag1—O3^{iv}	87.7 (7)	O6—Ag9—S2	85.6 (6)
O7^{iv}—Ag1—O3^{iv}	81.6 (9)	C17^{iii}—Ag10—S1	99.2 (19)
O3—Ag1—O16	105.9 (7)	C1^{iv}—Ag10—S1	131.3 (18)
O3—Ag1—O7^{iv}	96.0 (10)	C17^{iii}—Ag11—C36	152 (2)
O3—Ag1—O3^{iv}	158.1 (5)	C36—Ag11—Ag13	48.0 (10)
O3—Ag1—O5	28.5 (9)	S1—Ag12—C42	118.1 (7)
O5—Ag1—O1^{vi}	98.9 (7)	C36—Ag12—S1	97.5 (11)
O5—Ag1—O7^{iv}	86.7 (9)	C36—Ag12—C42	121.4 (15)
O5—Ag1—O3^{iv}	167.3 (9)	C36—Ag12—O6	115.9 (13)
C17—Ag2—C22	171 (2)	C41—Ag12—S1	134.5 (8)
C17—Ag2—C23	144.6 (19)	C41—Ag12—C36	123.4 (15)
C22—Ag2—C23	26.6 (13)	C41—Ag12—C42	24.8 (9)
C46—Ag3—C47	26.7 (7)	C41—Ag12—O6	95.8 (11)
C46 —Ag3—O9^{iv}	112.5 (10)	O6—Ag12—S1	81.5 (7)
O9^{iv}—Ag3—C47	139.1 (9)	O6—Ag12—C42	114.3(11)
O9^{vi}—Ag4—O5	118.0 (9)	O8—Ag13—O82	76.4 (15)
O9^{iv}—Ag4—O5	87.7 (10)	O8—Ag13—C31	106.7 (10)
O9^{iv}—Ag4—O9^{vi}	94.9 (17)	O8^{ii}—Ag13—C31	106.7 (10)
O9^{iv}—Ag4—C22	104.4 (12)	C36—Ag13—O8^{ii}	123.3 (12)
O9^{vi}—Ag4—C22	104.4 (12)	C36—Ag13—O8	123.3 (12)
C22—Ag4—O5	134.7 (14)	C36—Ag13—C31	114.2 (16)
C46—Ag5—S3	97.0 (8)	O2—Ag14—O8	105.3 (9)
C22—Ag5—C46	119.5 (12)	O2—Ag14—O3	42.8 (9)
C46—Ag6—O7	115.6 (9)	O1—Ag15—O2	95.5 (6)
C46—Ag6—O3	149.3(10)	C31—Ag15—O1	172.6 (12)
C46—Ag6—O5	142.7 (9)	C31—Ag15—O2	87.3 (7)
O3—Ag6—O7	87.3 (8)	O2—Ag16—C31	79.8 (9)
O3—Ag6—O5	29.2 (9)	O2—Ag16—C31^{i}	102.9 (9)
O5—Ag6—O7	101.4 (8)	C27—Ag16—C31^{i}	124.7 (8)
O7^{iv}—Ag7—O7	53.4 (15)	C27—Ag16—C31	124.7 (8)
C12—Ag7—O7^{iv}	153.3 (8)	C27—Ag16—O2	115.4 (14)
C12—Ag7—O7	153.3 (8)	S3—Ag17—O4	88.9 (4)

C12—Ag8—S2	110.4 (3)	C41—Ag17—S3	102.6 (9)
$C1^{iv}$—Ag8—S2	132.4 (19)	C41—Ag17—O4	120.7 (8)
$C1^{iv}$—Ag8—C12	112.9 (19)	C27—Ag17—S3	125.5 (13)
C46—Ag9—S2	103.3 (6)	C27—Ag17—O4	96.9 (10)
C46—Ag9—O6	119.3 (10)	C27—Ag17—C41	119.4 (13)
C41—Ag9—S2	128.0 (7)	C31—Ag16—$C31^{i}$	99.3 (12)
(i) 1/2-*x*, 3/2-*y*, +*z*; (ii) +*x*, 3/2-*y*,+*z*; (iii) 1-*y*, 1/2+*x*, 1/2-*z*; (iv) 1-*y*, 1-*x*,1/2-*z*; (v) 1/2-*x*,+*y*,+*z*; (vi) -1/2+*y*, 1-*x*, 1/2-*z*.			

Table S6: Selected bond distances (Å) and angles (°) for Ag104b·Ag108a.

Ag1—Ag1^{i}	2.75 (4)	Ag11—O11	2.512 (14)
Ag1—Ag4A^{ii}	3.37 (2)	Ag11—C60	2.17 (4)
Ag1—Ag7	2.89 (2)	Ag12—Ag27^{ii}	3.072 (3)
Ag1—Ag7^{i}	3.10 (2)	Ag12—O3^{ii}	2.237 (14)
Ag1—O9^{ii}	2.295 (17)	Ag12—O9	2.243 (12)
Ag1—C16	2.20 (3)	Ag12—C86^{ii}	2.11 (3)
Ag1—C16^{i}	2.261(3)	Ag13—Ag20^{ii}	2.878 (3)
Ag1—S1^{ii}	2.62 (3)	Ag13—Ag24	2.935 (3)
Ag2—Ag8	2.984 (5)	Ag13—S3	2.547 (8)
Ag2—Ag20	2.856 (3)	Ag13—C60	2.34 (4)
Ag2—S5	2.516 (7)	Ag13—C68	2.53 (4)
Ag2—C70	2.68 (4)	Ag13—C70^{ii}	2.35 (5)
Ag2—C94	2.37 (4)	Ag14—S5	2.567 (7)
Ag3—Ag17^{iii}	2.657 (5)	Ag14—O8	2.510 (13)
Ag3—Ag17	2.658 (5)	Ag14—C60^{iii}	2.46 (4)
Ag3—Ag18	3.206 (3)	Ag14—C86	2.39 (3)
Ag3—O1	2.480 (12)	Ag15—Ag23	3.012 (3)
Ag3—O3	2.463 (15)	Ag15—S3	2.493 (8)
Ag3—O11^{iii}	2.510 (15)	Ag15—O7	2.347 (17)
Ag4—Ag5A	2.974 (17)	Ag15—C93	2.37 (8)
Ag4—Ag7^{iii}	2.863 (11)	Ag16—Ag19^{ii}	3.047 (2)
Ag4—Ag16^{iii}	3.270(13)	Ag16—Ag26	3.023 (2)
Ag4—Ag18	3.175(11)	Ag16—Ag27	3.102 (3)
Ag4—Ag19	3.179 (14)	Ag16—S8	2.542 (6)
Ag4—Ag27^{iii}	2.986 (10)	Ag16—C62	2.31 (3)
Ag4—O1	2.418 (14)	Ag16—C11^{ii}	2.35 (4)
Ag4—O13^{iii}	2.57 (2)	Ag17—Ag23	3.052 (4)
Ag4—C11	2.33 (3)	Ag17—O6	2.251 (11)
Ag4—C16^{iii}	2.59 (3)	Ag17—O13	2.344 (14)
Ag5—Ag19	3.285 (17)	Ag17—O2	2.304 (14)
Ag5—Ag22	3.214 (17)	Ag17—O3	2.506 (14)
Ag5—Ag25	3.374 (13)	Ag18—Ag19	2.857 (2)
Ag5—C11	2.29 (5)	Ag18—Ag20	2.884 (3)
Ag5—S1	2.55 (2)	Ag18—Ag25	2.864 (3)
Ag6—Ag8	3.216 (12)	Ag18—O1	2.207 (13)
Ag6—Ag18	3.111 (12)	Ag18—O4	2.428 (13)
Ag6—Ag20	3.27 (2)	Ag18—O11^{iii}	2.525 (15)
Ag6—Ag23	3.12 (2)	Ag18—C80	2.05 (3)
Ag6—Ag25	3.073 (14)	Ag19—S2^{iii}	2.574 (7)
Ag6—C80	2.40 (4)	Ag19—C80	2.39 (4)
Ag6—C94	2.09 (4)	Ag19—C11	2.29 (4)

Ag6—O4	2.42 (2)	Ag20—Ag24^{iii}	2.874 (3)
Ag7—Ag7^{i}	3.012 (3)	Ag20—O8	2.403 (15)
Ag7—Ag12^{iii}	3.361 (2)	Ag20—O11^{iii}	2.319 (16)
Ag7—Ag17	3.125 (3)	Ag20—C70	2.07 (3)
Ag7—Ag27	2.899 (2)	Ag21—Ag23	2.909 (2)
Ag7—O2	2.097 (11)	Ag21—Ag25	2.840 (3)
Ag7—C16	2.029 (15)	Ag21—S4	2.548 (6)
Ag8—Ag26	3.044 (3)	Ag21—C56	2.30 (3)
Ag8—S6	2.623 (7)	Ag21—C60	2.37 (3)
Ag8—O10	2.395 (14)	Ag22—S4	2.548 (6)
Ag8—C94	2.10 (4)	Ag22—S1	2.331 (12)
Ag9—Ag12^{iii}	3.060 (2)	Ag22—O5	2.595 (12)
Ag9—Ag14	2.980 (2)	Ag22—C86^{ii}	2.55 (3)
Ag9—Ag26	3.285 (2)	Ag23—Ag25	3.135 (3)
Ag9—Ag27	3.027 (2)	Ag23—O4	2.363 (15)
Ag9—S6	2.580 (6)	Ag23—O6	2.462 (13)
Ag9—O10	2.420 (13)	Ag23—O7	2.308 (15)
Ag9—C86	2.31 (3)	Ag23—C56	1.99 (3)
Ag10—Ag15	2.888(4)	Ag24—S2	2.544 (6)
Ag10—Ag24	3.235 (3)	Ag24—S7	2.571 (7)
Ag10—Ag26	2.823 (3)	Ag24—C70^{ii}	2.35 (4)
Ag10—S7	2.521 (8)	Ag25—S9	2.544 (7)
Ag10—S8	2.580 (8)	Ag25—C56	2.33 (3)
Ag10—C93	2.57 (7)	Ag25—C80	2.43 (3)
Ag11—Ag12	3.307 (2)	Ag26—Ag27	2.910 (3)
Ag11—Ag13	3.088 (2)	Ag26—O10	2.423 (15)
Ag11—Ag14^{ii}	3.037 (2)	Ag26—C62	2.27 (4)
Ag11—Ag20^{ii}	3.310 (3)	Ag26—C93	2.46 (7)
Ag11—Ag21	2.905 (2)	Ag27—O13	2.291 (16)
Ag11—Ag23	3.122 (3)	Ag27—C16	2.50 (3)
Ag11—O6	2.355 (12)	Ag27—C62	2.20 (3)
O9^{ii}—Ag1—S1^{ii}	100.8 (7)	C86—Ag14—O8	117.8 (8)
O9^{ii}—Ag1—C16^{i}	81.9 (8)	O7—Ag15—S3	119.4 (5)
C16^{i}—Ag1—S1^{ii}	113.2 (7)	C93—Ag15—S3	129 (2)
C16—Ag1—S1^{ii}	122.9 (9)	C93—Ag15—O7	103.3 (17)
C16—Ag1—O9^{ii}	121.0 (9)	C62—Ag16—C89^{ii}	113.2 (12)
S5—Ag2—C70	105.8 (11)	C62—Ag16—S8	105.7 (9)
C94—Ag2—S5	129.7 (9)	C62—Ag16—O12	117.5 (9)
C94—Ag2—C70	121.7 (14)	C62—Ag16—C11^{ii}	118.9 (12)
O1—Ag3—O11^{iii}	86.2 (4)	C11^{ii}—Ag16—S8	121.0 (9)
O1—Ag3—O1	118.3 (5)	C11^{ii}—Ag16—C89^{ii}	22.9 (10)
O3—Ag3—O11^{iii}	93.8 (5)	O6—Ag17—O13	110.2 (5)
O1—Ag4—O13^{iii}	83.9 (5)	O6—Ag17—O2	110.3 (5)

O1—Ag4—C16^{iii}	112.9 (7)	O6—Ag17—O3	142.7 (5)
O13^{iii}—Ag4—C16^{iii}	93.9 (8)	O13—Ag17—O3	96.4 (5)
C11—Ag4—O1	135.2 (12)	O2—Ag17—O13	102.0 (5)
C11—Ag4—O13^{iii}	112.4 (12)	O2—Ag17—O3	88.2 (5)
C11—Ag4—C16^{iii}	107.4 (11)	O1—Ag18—O4	94.7 (4)
S1—Ag5—C89	103.5 (9)	O1—Ag18—O11^{iii}	91.9 (5)
C11—Ag5—S1	114.7 (11)	O4—Ag18—O11^{iii}	89.7 (5)
C11—Ag5—C89	22.8 (10)	C80—Ag18—O1	154.0 (10)
O4—Ag6—C78	113.9 (11)	C80—Ag18—O4	99.7 (11)
C80—Ag6—O4	90.8 (10)	C80—Ag18—O11^{iii}	109.5 (10)
C80—Ag6—C78	25.1 (9)	C80—Ag19—S2^{ii}	94.1 (7)
C94—Ag6—O4	117.1 (11)	C11—Ag19—S2^{iii}	120.8 (10)
C94—Ag6—C78	113.0 (15)	C11—Ag19—C80	140.4 (13)
C94—Ag6—C80	134.5 (17)	O11^{iii}—Ag20—O8	82.5 (5)
C16 —Ag7—O2	173.9 (9)	C70—Ag20—O8	111.8 (13)
O10—Ag8—S6	84.6 (3)	C70—Ag20—O11^{ii}	140.9 (15)
C94—Ag8—S6	135.0 (10)	C56—Ag21—S4	133.7 (13)
C94—Ag8—O10	120.6 (10)	C56—Ag21—C60	123.8 (16)
O10—Ag9—S6	85.0 (4)	C60—Ag21—S4	96.1 (9)
O10—Ag9—C62	84.0 (8)	S4—Ag22—O5	88.3 (3)
C62—Ag9—S6	100.5 (8)	S1—Ag22—S4	127.7 (3)
C86—Ag9—S6	132.0 (8)	S1—Ag22—O5	94.6 (4)
C86—Ag9—O10	118.3 (8)	C86^{iii}—Ag22—S4	108.3 (8)
C86—Ag9—C62	121.9 (12)	C86^{iii}—Ag22—O5	121.6 (7)
S7—Ag10—S8	101.2 (2)	O4—Ag23—O6	94.0 (4)
O6—Ag11—O11	85.7 (5)	O7—Ag23—O4	104.8 (6)
C60—Ag11—O6	137.2 (10)	O7—Ag23—O6	92.2 (5)
C60—Ag11—O11	131.0 (10)	C56—Ag23—O4	111.5 (14)
O3^{ii}—Ag12—O9	90.8 (4)	C56—Ag23—O6	131.0 (15)
C86^{ii}—Ag12—O9	136.9 (9)	C56—Ag23—O7	118.5 (15)
C86^{iii}—Ag12—O3^{ii}	132.1 (9)	S2—Ag24—S7	100.8 (2)
C60—Ag13—S3	96.8 (9)	C70^{ii}—Ag24—S2	119.8 (12)
C60—Ag13—C68	27.8 (10)	C70^{ii}—Ag24—S7	122.1 (11)
C60—Ag13—C70^{ii}	123.1 (15)	C56—Ag25—S9	138.0 (13)
C68—Ag13—S3	96.8 (9)	C56—Ag25—C80	110.5 (15)
C70^{ii}—Ag13—S3	134.9 (10)	C80—Ag25—S9	102.8 (8)
C70^{ii}—Ag13—C68	119.2 (14)	C62—Ag26—O10	90.6 (9)
O8—Ag14—S5	90.4 (4)	C62—Ag26—C93	151 (2)
C60^{ii}—Ag14—S5	99.6 (8)	C93—Ag26—O10	105.7 (19)
C60^{ii}—Ag14—O8	88.3 (9)	O13—Ag27—C16	103.9 (6)
C86—Ag14—S5	128.7 (7)	C62—Ag27—O13	117.7 (10)
C86—Ag14—C60^{iii}	122.1 (11)	C62—Ag27—C16	110.8 (11)
(i) 1-*x*, 1-*y*, +*z*; (ii) +*y*, 1-*x*, 1-*z*; (iii) 1-*y*,+*x*, 1-*z*.			

Table S7: Selected bond distances (Å) and angles (°) for Ag104c·Ag108b.

Ag1—Ag11^{i}	2.864 (12)	Ag15—Ag19	3.084 (5)
Ag1—Ag13	3.114 (12)	Ag15—Ag20	3.150 (7)
Ag1—Ag25^{i}	3.339 (13)	Ag15—Ag21	2.885 (5)
Ag1—Ag26^{i}	2.892 (3)	Ag15—O1	2.50 (2)
Ag1—S3^{i}	2.45 (2)	Ag15—O9	2.37 (3)
Ag1—S8	2.655 (18)	Ag15—O11^{iii}	2.13 (3)
Ag1A—Ag11^{i}	3.156 (16)	Ag15—C52	2.14 (8)
Ag1A—S8	2.44 (2)	Ag16—Ag22	2.926 (6)
Ag2—Ag3	3.111 (13)	Ag16—Ag24^{i}	2.942 (6)
Ag2—Ag15	2.822 (10)	Ag16—O4	2.38 (3)
Ag2—Ag19	3.116 (10)	Ag16—O6	2.32 (3)
Ag2—O9	2.39 (3)	Ag16—C14	2.70 (7)
Ag2—C32	2.11 (5)	Ag16—C53	1.94 (9)
Ag2—C52	2.38 (8)	Ag16—C101	2.28 (8)
Ag2A—Ag3A	3.134 (17)	Ag17—Ag18	3.018 (5)
Ag2A—Ag4A	3.288 (16)	Ag17—Ag22	2.844 (5)
Ag2A—Ag16	3.021 (17)	Ag17—Ag25^{i}	3.084 (5)
Ag2A—O9	2.52 (3)	Ag17—S9	2.553 (14)
Ag2A—C14	2.16 (7)	Ag17—C14	2.46 (7)
Ag3—Ag4	3.035 (15)	Ag17—C30	2.29 (7)
Ag3—Ag26	3.012 (9)	Ag18—Ag22	3.140 (5)
Ag3—S2	2.77 (2)	Ag18—Ag23^{i}	3.008 (5)
Ag3—O5	2.02 (3)	Ag18—Ag25^{i}	3.141 (5)
Ag3—C32	2.11 (6)	Ag18—O12	2.36 (2)
Ag3A—Ag11	2.882 (15)	Ag18—O13	2.45 (3)
Ag3A—C32	2.17 (7)	Ag18—C30	2.10 (7)
Ag4—Ag16	2.886 (10)	Ag19—Ag21	2.865 (5)
Ag4—S5	2.493 (16)	Ag19—Ag22	2.920 (5)
Ag4—O4	2.50 (3)	Ag19—S6	2.552 (13)
Ag4—C32	2.35 (6)	Ag19—C14	2.48 (6)
Ag4A—S5	2.460 (17)	Ag17—C30	2.29 (7)
Ag4A—C32	2.00 (6)	Ag19—Ag21	2.865 (5)
Ag5—Ag5^{ii}	3.116 (6)	Ag19—Ag22	2.920 (5)
Ag5—Ag10	3.341(6)	Ag18—Ag23^{i}	3.008(5)
Ag5—Ag10^{ii}	3.125 (6)	Ag18—Ag25^{i}	3.141 (5)
Ag5—Ag18^{iii}	2.957 (5)	Ag18—O12	2.36 (2)
Ag5—Ag20	3.204 (7)	Ag18—O13	2.45 (3)
Ag5—Ag23	2.947 (4)	Ag18—C30	2.10 (7)
Ag5—O2	2.06 (2)	Ag18—Ag22	3.140 (5)
Ag5—O8^{i}	2.50 (3)	Ag19—S6	2.552 (13)
Ag5 —C41	2.06 (6)	Ag19—C14	2.48 (6)

Ag6—Ag7^{iii}	3.144 (6)	Ag19—C52	2.22 (7)
Ag6—Ag17	3.320 (6)	Ag20—As3	3.106 (8)
Ag6—Ag18	2.947 (5)	Ag20—O1	2.26 (2)
Ag6—S6	2.579 (15)	Ag20—O2	2.31 (3)
Ag6—S7^{iii}	2.523 (19)	Ag20—O3	2.36 (3)
Ag6—O10	2.45 (2)	Ag20—O13^{iii}	2.34 (3)
Ag6—C30	2.32 (7)	Ag21—S4	2.552 (13)
Ag7—Ag27	2.926 (5)	Ag21—C46	2.23 (6)
Ag7—S4^{i}	2.529 (15)	Ag21—C52	2.42 (7)
Ag7—S7	2.402 (16)	Ag20—As3	3.106 (8)
Ag7—O10^{i}	2.48 (2)	Ag20—O1	2.26 (2)
Ag7—C50	2.47 (7)	Ag20—O2	2.31 (3)
Ag8—Ag20	2.700 (9)	Ag20—O3	2.36 (3)
Ag8—Ag20^{iii}	2.704 (9)	Ag20—O13^{iii}	2.34 (3)
Ag8—Ag22^{iii}	3.258 (5)	Ag21—S4	2.552 (13)
Ag8—O1	2.58 (2)	Ag21—C46	2.23 (6)
Ag8 —O3^{iii}	2.46 (3)	Ag21—C52	2.42 (7)
Ag8—O6^{iii}	2.45 (3)	Ag22—O6	2.54 (3)
Ag8—O12^{iii}	2.54 (2)	Ag22—O9	2.41 (3)
Ag9—Ag14^{iii}	3.036 (5)	Ag22—O12	2.22 (3)
Ag9—Ag15	3.115 (5)	Ag22—C14	2.03 (7)
Ag9—Ag16^{iii}	3.360 (6)	Ag23—Ag25	3.085 (5)
Ag9—Ag21	2.953 (5)	Ag23—Ag26	2.937 (5)
Ag9—Ag24	3.045 (5)	Ag23—Ag27	3.076 (5)
Ag9—O1	2.29 (2)	Ag23—O13^{iii}	2.33 (3)
Ag9—O6^{iii}	2.43 (3)	Ag23—C76	2.19 (5)
Ag9—C46	2.07 (4)	Ag24—S1	2.498 (15)
Ag10—Ag10^{ii}	3.133 (11)	Ag24—C46	2.49 (6)
Ag10—Ag18^{i}	3.152 (6)	Ag24—C53^{iii}	2.34 (17)
Ag10—S7	2.415 (18)	Ag25—Ag26	2.999 (5)
Ag10—O8^{i}	2.29 (2)	Ag25—S8^{iii}	2.565 (12)
Ag10—C41^{ii}	2.23 (6)	Ag25—O7^{iii}	2.46 (3)
Ag11—Ag15	3.127 (6)	Ag25—C30^{iii}	2.44 (7)
Ag11—S1	2.450 (17)	Ag25—C48^{iii}	2.69 (7)
Ag11—O11^{iii}	2.36 (3)	Ag25—C76	2.36 (7)
Ag11—C3	2.27 (7)	Ag26—O5	2.38 (3)
Ag12—Ag14	3.016 (5)	Ag26—C3	2.22 (10)
Ag12—Ag23	3.014 (5)	Ag26—C23	2.22 (10)
Ag12—Ag26	3.339 (5)	Ag26—C76	2.12 (6)
Ag12—Ag27	3.001 (5)	Ag27—O3	2.35 (3)
Ag12—S2	2.525 (15)	Ag27—O8^{i}	2.33 (3)
Ag12—O5	2.55 (3)	Ag27—C50	2.09 (4)
Ag12—C50	2.39 (7)	As1—O2^{i}	1.66 (2)

Ag13—Ag16	2.898 (6)	As1—O2^{ii}	1.66 (2)
Ag13—Ag24^{i}	2.978 (5)	As2—O6	1.74 (2)
Ag13—S3^{i}	2.576 (13)	As2—O7	1.57 (3)
Ag13—S9	2.544 (12)	As2—O11	1.65 (3)
Ag13—C53	2.26 (19)	As2—O13	1.70 (3)
Ag14—Ag27	2.977 (5)	As3—O3	1.68 (3)
Ag14—S5	2.543 (14)	As3—O4	1.71 (3)
Ag14—O4	2.52 (3)	As3—O5	1.75 (3)
Ag14—C46^{i}	2.48 (6)	As3—O9	1.71 (3)
Ag14—C50	2.33 (7)	As4—O1	1.71 (2)
S3^{i}—Ag1—S8	102.6 (6)	As4—O8	1.71 (3)
S3^{i}—Ag1—O7	111.9 (8)	As4—O10	1.71 (2)
O7—Ag1—S8	87.3 (7)	As4—O12	1.70 (3)
S8—Ag1A—S3^{i}	104.7 (7)	C101—Ag16—C14	120 (3)
S8—Ag1A—C3^{i}	136 (2)	C14—Ag17—S9	92.5 (14)
C3^{i}—Ag1A—C97^{i}	28 (2)	C30—Ag17—S9	125.9 (18)
C32—Ag2—O9	115.0 (17)	C30—Ag17—C14	139 (2)
C52—Ag2—O9	98.5 (18)	O12—Ag18—O13	82.0 (9)
C14—Ag2A—O9	90 (2)	O12—Ag18—C41^{i}	107.6 (14)
C14—Ag2A—C16	32 (2)	C30—Ag18—O12	135.8 (19)
C14—Ag2A—C32	153 (2)	C30—Ag18—O13	125.1 (19)
C16—Ag2A—O9	122.5 (19)	C14—Ag19—S6	102.0 (17)
C32—Ag2A—C16	127 (2)	C52—Ag19—S6	134 (2)
C32—Ag2A—C54	27 (2)	C52—Ag19—C14	111 (3)
O5—Ag3—S2	85.7 (8)	O1—Ag20—O2	112.8(9)
S5—Ag4—O4	88.8 (7)	O1—Ag20—O3	151.0 (9)
C32—Ag4—S5	130.2 (16)	O2—Ag20—O3	85.5 (10)
O2—Ag5—O8^{i}	94.5 (9)	O2—Ag20—O13^{iii}	95.3 (9)
C4^{i}—Ag5—O2	177 (2)	O13^{iii}—Ag20—O3	93.3 (10)
C41—Ag5—O8^{i}	87.3 (19)	C46—Ag21—S4	98.5 (15)
S7^{iii}—Ag6—S6	124.6 (5)	C46—Ag21—C52	127 (2)
O10—Ag6—S6	89.8 (7)	C52—Ag21—S4	128 (2)
O10—Ag6—S7^{iii}	91.8 (7)	O9—Ag22—O6	88.5 (9)
C30—Ag6—S6	112.0 (17)	O12—Ag22—O6	91.2 (9)
C30—Ag6—S7^{iii}	110.1 (17)	O12—Ag22—O9	97.5 (9)
C30—Ag6—O10	127.7 (19)	C14—Ag22—O9	97 (2)
S7—Ag7—S4^{i}	125.2 (5)	C14—Ag22—O12	155 (2)
S7—Ag7—O10^{i}	94.0 (7)	O13^{iii}—Ag23—C41	98.2 (16)
S7—Ag7—C50	113.6 (18)	C76—Ag23—O13^{iii}	121.5 (18)
O10^{i}—Ag7—S4^{i}	87.4 (6)	C76—Ag23—C41	115 (2)
C50—Ag7—O10^{i}	125.2 (10)	C46—Ag24—S1	97.6 (14)
O3^{iii}—Ag8—O1	88.2 (9)	C53^{iii}—Ag24—S1	135 (4)
O6^{iii}—Ag8—O1	76.1 (8)	C53^{iii}—Ag24—C46	124 (5)

O6^{iii}—Ag8—O3^{iii}	93.2 (9)	C101^{iii}—Ag24—S1	135 (2)
O6^{iii}—Ag8—O12^{iii}	86.2 (8)	C101^{iii}—Ag24—C46	118 (3)
O12^{iii}—Ag8—O1	149.6 (8)	C101^{iii}—Ag24—C53^{iii}	13 (4)
O1—Ag9—O6^{iii}	82.1 (8)	S8^{iii}—Ag25—C48^{iii}	104.3 (15)
C46—Ag9—O1	135.7 (17)	O7 ^{iii}—Ag25—S8^{iii}	91.9 (7)
C46—Ag9—O6^{iii}	137.3 (18)	O7^{iii}—Ag25—C48^{iii}	116.0 (17)
O8^{i}—Ag10—S7	108.1 (7)	C30^{iii}—Ag25—S8^{iii}	121.8 (17)
C41^{ii}—Ag10—S7	128.3 (16)	C30^{iii}—Ag25—O7^{iii}	96.4 (18)
C41^{ii}—Ag10—O8^{i}	112.9 (17)	C30^{iii}—Ag25—C48^{iii}	24.5 (19)
O11^{iii}—Ag11—S1	111.3 (8)	C76—Ag25—S8^{iii}	106.0 (16)
C3—Ag11—S1	139 (3)	C76—Ag25—O7^{iii}	115.4 (15)
C3—Ag11—O11^{iii}	104 (2)	C76—Ag25—C30^{iii}	121 (2)
C23—Ag11—S1	137 (3)	C76—Ag25—C48^{iii}	118 (2)
C23—Ag11—O11^{iii}	104 (2)	C3—Ag26—O5	96 (3)
S2—Ag12—O5	81.3 (8)	C23—Ag26—O5	100 (3)
C50—Ag12—C76	127 (2)	C76—Ag26—O5	95.1 (19)
C50—Ag12—S2	129.5 (16)	C76—Ag26—C3	157 (3)
S3^{i}—Ag13—C101	117 (2)	C76—Ag26—C23	155 (3)
S9—Ag13—S3^{i}	101.1 (4)	O8^{i}—Ag27—O3	87.7 (9)
S9—Ag13—C101	128 (2)	C50—Ag27—O3	137.7 (19)
C53—Ag13—S3^{i}	124 (3)	C50—Ag27—O8^{i}	134.5 (19)
C53—Ag13—S9	122 (4)	O2—As1—O2^{iii}	111.3 (9)
O4—Ag14—S5	87.2 (7)	O2—As1—O2^{i}	105.8 (16)
C46^{i}—Ag14—S5	104.7 (11)	O2—As1—O2^{ii}	111.3 (9)
C46^{i}—Ag14—O4	87.8 (15)	O7—As2—O6	118.6 (13)
C50—Ag14—S5	128.7 (13)	O7—As2—O11	114.1 (14)
C50—Ag14—O4	120.1 (15)	O7—As2—O13	107.4 (14)
C50—Ag14—C46^{i}	118 (2)	O11—As2—O6	105.1 (13)
O9—Ag15—O1	94.1(9)	O11—As2—O13	109.6 (14)
O11^{iii}—Ag15—O1	86.5 (9)	O13—As2—O6	101.1 (13)
O11^{iii}—Ag15—O9	106.9 (10)	O3—As3—O4	109.9 (14)
O11^{iii}—Ag15—C52	121.7 (19)	O3—As3—O5	115.1 (14)
C52—Ag15—O1	136 (2)	O3—As3—O9	106.7 (13)
C52—Ag15—O9	106 (2)	O4—As3—O5	108.1 (14)
O4—Ag16—C14	118.5 (15)	O4—As3—O9	108.9 (13)
O6—Ag16—O4	79.1 (9)	O9—As3—O5	108.0 (13)
O6—Ag16—C14	96.0 (15)	O1—As4—O10	110.5 (12)
C53—Ag16—O4	112 (6)	O8—As4—O1	110.7 (12)
C53—Ag16—O6	138 (5)	O8—As4—O10	108.2 (12)
C53—Ag16—C14	111 (5)	O12—As4—O1	110.9 (12)
C101—Ag16—O4	101 (3)	O12—As4—O8	106.0 (12)
C101—Ag16—O6	137 (3)	O12—As4—O10	110.5 (12)
(i) 1+*y*, 1-*x*, 1-*z*; (ii) 1-*x*, 1-*y*, +*z*; (iii) 1-*y*,+*x*, 1-*z*.			

Reference:

1. Zhao, L.; Wan, C.-Q.; Han, J.; Chen, X.-D.; Mak, T. C. W. Ancillary Ligands and Spectator Cations as Controlling Factors in the Construction of Coordination and Hydrogen-Bonded Networks with the ^{t}BuC≡CAg$_n$ (n=4, 5) Supramolecular Synthon. *Chem. Eur. J.* **2008**, *14*, 10437-10444.
2. Wagner, J.; Ciesielski, M.; Fleckenstein, C. A.; Denecke, H.; Garlichs, F.; Ball, A.; Doering, M. Benign and High-Yielding, Large-Scale Synthesis of Diphenylphosphinodithioic Acid and Related Compounds. *Org. Process Res. Dev.* **2013**, *17*, 47-52.
3. Palatinus L. Chapuis G. SUPERFLIP - A Computer Program for the Solution of Crystal Structures by Charge Flipping in Arbitrary Dimensions *J. Appl. Crystallogr*. **2007**, *40*, 786-790.
4. Sheldrick, G. M. SHELXT-Integrated Space-Group and Crystal-Structure Determination. *Acta Cryst*. **2015**, *A71*, 3-8.
5. Dolomanov, O. V.; Bourhis, L. J.; Gildea, R. J.; Howard, J. A. K.; Puschmann, H. OLEX2: A Complete Structure Solution, Refinement and Analysis Program. *J. Appl. Crystallogr*. **2009**, *42*, 339-341.
6. Spek, A. L. Structure Validation in Chemical Crystallography. *Acta Crystallogr., D: Biol. Crystallogr*. **2009**, *65*, 148-155.
7. Amsterdam Density Functional (ADF2019) Code, Vrije Universiteit: Amsterdam, The Netherlands.
8. van Lenthe, E.; Baerends, E.-J. J.; Snijders, J. G. Relativistic Total Energy Using Regular Approximations. *J. Chem. Phys.* **1994**, *101*, 9783.
9. Perdew, J. P.; Burke, K.; Wang, Y. Generalized Gradient Approximation for the Exchange-Correlation Hole of a Many-Electron System. *Phys. Rev. B.* **1996**, *54*, 16533–16539.
10. Perdew, J. P.; Burke, K.; Ernzerhof, M. Generalized Gradient Approximation Made Simple. *Phys. Rev. Lett.* **1997**, *78*, 1396–1396.
11. Ehrlich, S.; Moellmann, J.; Grimme, S. Dispersion-Corrected Density Functional Theory for Aromatic Interactions in Complex Systems. *Acc. Chem. Res.* **2013**, *46*, 916–926.
12. Grimme, S. Density Functional Theory with London Dispersion Corrections. *Wiley Interdiscip. Rev. Comput. Mol. Sci.* **2011**, *1*, 211–228.
13. Zhang, W.; Liu, Z.; Song, K.; Aikens, C. M.; Zhang, S.; Wang, Z.; Tung, C.; Sun, D. A 34-

Electron Superatom Ag78 Cluster with Regioselective Ternary Ligands Shells and Its 2D Rhombic Superlattice Assembly. *Angew. Chem. Int. Ed.* **2021**, *60*, 4231–4237.

14. Wang, Z.; Alkan, F.; Aikens, C. M.; Kurmoo, M.; Zhang, Z.; Song, K.; Tung, C.; Sun, D. An Ultrastable 155-Nuclei Silver Nanocluster Protected by Thiacalix[4]Arene and Cyclohexanethiol for Photothermal Conversion. *Angew. Chem. Int. Ed.* **2022**, *61*, e202206742.
15. Weerawardene, K. L. D. M.; Aikens, C. M. Theoretical Insights into the Origin of Photoluminescence of $Au_{25}(SR)_{18}^{-}$ Nanoparticles. *J. Am. Chem. Soc.* **2016**, *138*, 11202–11210.
16. Ebina, M.; Iwasa, T.; Harabuchi, Y.; Taketsugu, T. Time-Dependent Density Functional Theory Study on Higher Low-Lying Excited States of $Au_{25}(SR)_{18}^{-}$. *J. Phys. Chem. C* **2018**, *122*, 4097–4104.
17. Versluis, L.; Ziegler, T. The Determination of Molecular Structures by Density Functional Theory. The Evaluation of Analytical Energy Gradients by Numerical Integration. *J. Chem. Phys.* **1988**, *88*, 322–328.
